# How to Improve Cognitive Flexibility: Evidence From Noninvasive Neuromodulation Techniques

**DOI:** 10.1111/cns.70613

**Published:** 2025-09-16

**Authors:** Naeimeh Akbari Gharalari, Solmaz Fallahi, Elnaz Nakhjiri, Leila Hosseini, Saba Pakkhou, Naser Havaei, Sina Khodakarimi, Parviz Shahabi, Mohsen Jafarzadeh Gharehziaaddin, Hamid Soltani Zangbar

**Affiliations:** ^1^ Neurophysiology Research Center, Cellular and Molecular Medicine Research Institute Urmia University of Medical Sciences Urmia Iran; ^2^ Department of Pharmacology and Toxicology, Faculty of Pharmacy Tabriz University of Medical Sciences Tabriz Iran; ^3^ Department of Physiology, Faculty of Medicine Tabriz University of Medical Sciences Tabriz Iran; ^4^ Research Center of Psychiatry and Behavioral Sciences Tabriz University of Medical Sciences Tabriz Iran; ^5^ Department of Medical Informatics, School of Management and Medical Informatics Tabriz University of Medical Sciences Tabriz Iran; ^6^ Department of Occupational Therapy, Faculty of Rehabilitation Sciences Tabriz University of Medical Sciences Tabriz Iran; ^7^ Department of Neurosciences and Cognition, Faculty of Advanced Medical Sciences Tabriz University of Medical Sciences Tabriz Iran; ^8^ Department of Languages and Cultures University of Aveiro, Campus Universitario de Santiago Aveiro Portugal

**Keywords:** cognitive flexibility, neurofeedback, noninvasive neuromodulation, photobiomodulation, rTMS, tDCS, virtual reality

## Abstract

**Background:**

Cognitive flexibility (CF) is a core component of executive function that enables humans to adaptively process and respond effectively to diverse and dynamic contextual needs. The execution of CF‐related tasks depends on the collective interaction of various neural circuits in essential brain regions, such as the prefrontal, anterior cingulate, and posterior parietal cortices, which support the harmonic and smooth transition of thoughts and perspectives. Therefore, clarifying the mechanism of CF and introducing non‐invasive neuromodulatory methods that enhance CF provides a way for developing new treatment approaches.

**Methods:**

This review systematically investigates the application of photobiomodulation (PBM), neurofeedback (NF), repetitive transcranial magnetic stimulation (rTMS), transcranial direct current stimulation (tDCS), and virtual reality (VR) as non‐invasive neuromodulatory techniques for modulating cognitive behaviors related to CF in neuropsychological conditions.

**Results:**

Considering the vital role of CF in executive functions, deficits in CF impair adaptive behavior in neuropsychological conditions such as schizophrenia, autism spectrum disorder (ASD), and age‐related cognitive decline. Some technology‐based interventions, including PBM, NF, rTMS, tDCS, and VR, have been developed to non‐invasively modulate the switching capability and strategies of the mind. These non‐invasive interventions provide targeted neuromodulation on neural circuits and have demonstrated a promising effect in improving cognitive processes, including CF.

**Conclusion:**

The present study provides a comprehensive review of the therapeutic consequences of non‐invasive neuromodulatory methods on CF in neurological conditions. This insight can offer a perspective for planning various research strategies and clinical approaches aimed at improving CF.

## Graphical abstract

In specific neuromodulatory techniques, such as tDCS, rTMS, and PBM, direct biophysical stimulations, including electrical, magnetic, or photonic stimulation, can enhance cognitive flexibility by modulating LTP, LTD, ATP, and synaptic plasticity. Cognitive flexibility can also be achieved through indirect, cognitive‐interactive approaches, like NF and VR techniques, which promote synaptic plasticity, neural connectivity, and regulation of neural interactions.

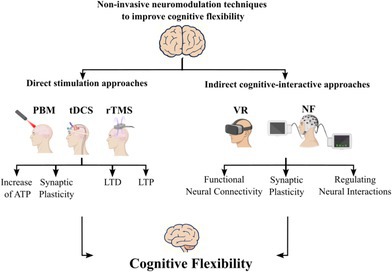

## 1 Introduction

Cognitive flexibility (CF) refers to the behavioral shifting in response to context demands and adaptive reaction to both internal and external needs [1]. Given the importance of appropriately responding to a dynamic and evolving environment, the cognitive system engages some feedback mechanisms to provide the best response at the optimal time [2]. CF induces transient changes in neural circuits and enables individuals to switch between two or more perspectives [3]. These feedback‐based reactions are supported by specific neural networks and brain regions connected through white matter tracts [4]. The CF encompasses both cognitive and behavioral components and plays a vital role in mental maturation, appropriate employment or educational procedures, and, finally, achieving a suitable social status [5]. This vital component of executive function depends on complicated neural circuits in different regions of the brain, including the prefrontal cortex (PFC), anterior cingulate cortex (ACC), posterior parietal cortex (PPC), and basal ganglia that significantly affect decision‐making, as a prerequisite for CF, through mental switching [6]. Various scales have been developed to assess CF, like the Cognitive Flexibility Scale and the Cognitive Control and Flexibility Questionnaire (CCFQ) [7]. Also, event‐related potential (ERP) studies, by designing appropriate switching paradigm tasks, evaluate some specific components' amplitude and latency, like p600 and N400 [8]. The CF is important even in animal models, and using experimental set‐shifting attention tasks, CF is examined in different conditions to measure the therapeutic consequences of different interventions [9, 10]. During typical neurodevelopment, the refinement of functional connectivity among brain regions supports the emergence of CF; however, abnormalities in this process—either in the form of excessive variability or weakened integration—are associated with CF dysfunction, as seen in neurodevelopmental disorders such as autism spectrum disorder (ASD) and neurodegenerative conditions like Alzheimer's disease (AD) [11]. Age‐related decline in CF further confirms its role in adaptive strategies, as inflexibility often coexists with broader executive dysfunctions in older adults [12]. Given the importance of the CF role in daily performance and executive functions, various treatment approaches have been investigated to improve it [13]. Nonpharmacological and noninvasive treatment approaches have gained growing attention due to their potential efficacy on CF in various neurological conditions and minimal side effects [14]. Noninvasive interventions, such as trans cranial direct current stimulation (tDCS), photobiomodulation (PBM), repetitive transcranial magnetic stimulation (rTMS), neurofeedback (NF), and virtual reality (VR), improve cognitive function by affecting and balancing cortical excitatory or inhibitory connections, modulating cortical excitability, enhancing synaptic plasticity, altering local blood flow, and importantly modulating the brain neural networks [15, 16, 17, 18, 19]. These technological approaches may enhance CF via reshaping neural connections and remapping the switching‐related networks. This review aims to summarize recent findings on the effectiveness of noninvasive neuromodulation techniques in improving CF.

## 2 Description of CF

CF involves the integration of external evidence with previous expectancies that lead to behavioral adjustment in response to changing environmental demands [20, 21, 22]. The CF includes three crucial components: the process of learning through cognitive switching, the development of adaptation strategies, and flexible response to unexpected alterations of context [8]. This cognitive construct engages several neurocognitive procedures, such as attention, executive functions, task switching, and inhibition [23]. The dynamic balance and interplay among these procedures are not fixed and vary among individuals [24]. From the psychological standpoint, CF is entangled with higher‐order cognitive mechanisms related to behavior control, such as inhibition and updating [3]. This regulatory mechanism creates a setting for behavioral adjustment in response to changes in internal and contextual cues, such that greater flexibility improves life quality, social communications, and age‐related cognitive decline [5, 25]. In contrast, cognitive rigidity is the inability to accommodate new conditions or situations, which is related to maladaptive states, like rumination and excessive worry [26]. CF is also influenced by factors such as cognitive reserve [27]. Cognitive reserve defines the brain's capacity to adapt and provide alternative mechanisms for task completion despite the cognitive side effects of aging or injury, such as those caused by AD or a stroke [28]. As a compensatory mechanism, individuals with greater cognitive reserve effectively manage neurological issues by recruiting alternative neural strategies prior to exhibiting apparent symptoms [29]. Cognitive reserve depends on the brain's capacity to generate enhanced, unique, adaptive, and flexible responses to the cognitive demands of the context. These flexible and reactive neural patterns explain the protective influence of cognitive reserve on cognitive decline [30]. The next part will describe the neural networks and brain regions involved in the CF process.

## 3 Neural Substrates and Networks Underlying CF

**FIGURE 1:**
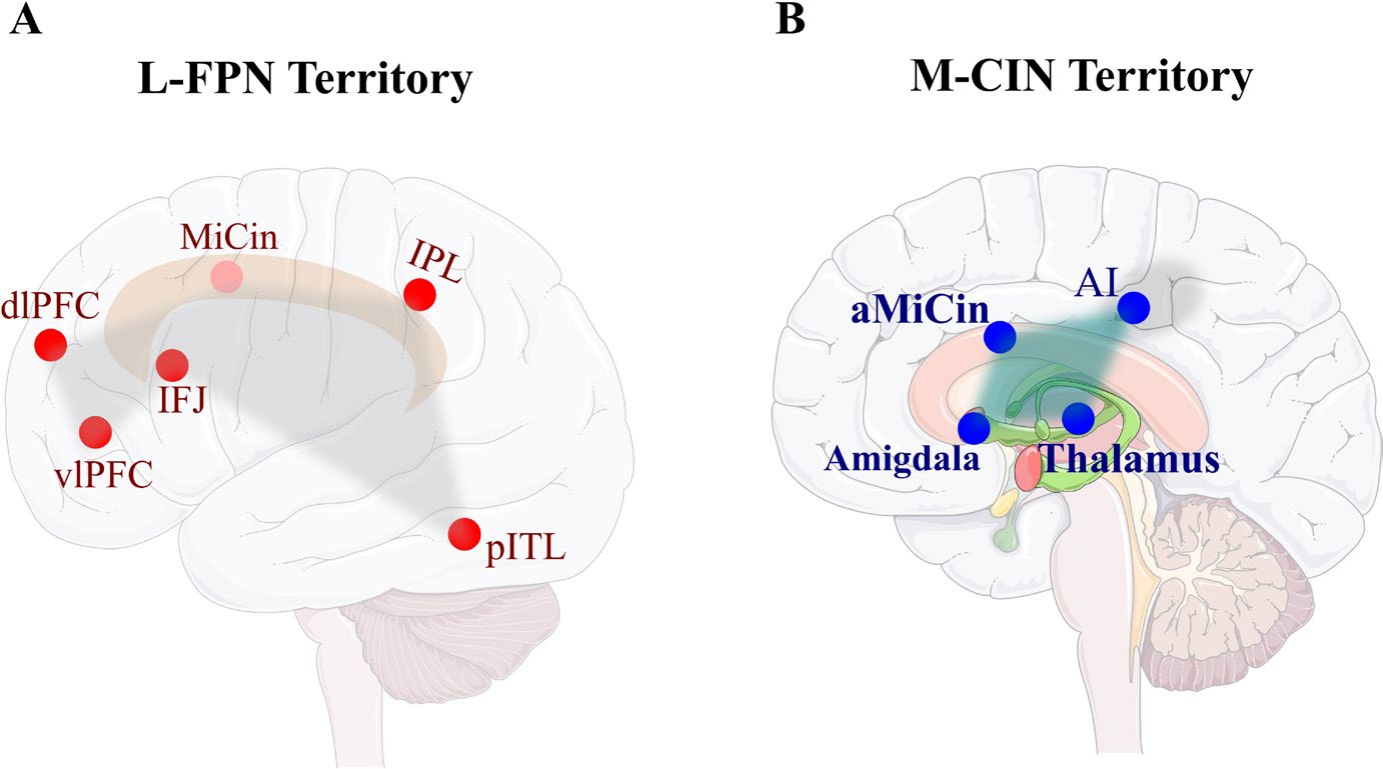
Territory of the lateral frontoparietal network (L‐FPN) and the midcingulo‐insular network (M‐CIN) in Cognitive flexibility. (A) The L‐FPN comprises lateral prefrontal cortices (PFCs); dorsolateral PFC (dlPFC), ventrolateral PFC, inferior frontal junction (IFJ), inferior parietal lobule (IPL), posterior inferior temporal lobes (pITL), and portions of the midcingulate (MiCin) gyrus. (B) The M‐CIN comprises bilateral anterior insulae (AI), the anterior midcingulate (aMiCin) cortex, and subcortical nodes, including the amygdala and thalamus.

The neural basis of CF has been widely investigated using set‐shifting paradigms, while the activity of brain regions is examined by functional MRI (fMRI) [31]. A wide range of CF‐related studies highlights the crucial function of the lateral frontoparietal network (L‐FPN) (Figure 1A) and the midcingulo‐insular network (M‐CIN) (Figure 1B) through task switching paradigms [31, 32]. The L‐FPN comprises key regions like dorsolateral PFC (dlPFC), ventrolateral PFC (vlPFC), the inferior frontal junction (IFJ), the inferior parietal lobule (IPL), posterior inferior temporal lobes (pITL), and the midcingulate (MiCin) gyrus. The M‐CIN includes key regions such as the cingulo‐opercular network and consists of bilateral anterior insulae (AI), the anterior midcingulate (aMiCin) cortex, the amygdala, and the thalamus [33].

Task‐dependent network connectivity verifies how specific task demands are related to dynamic representations and connections between brain portions, including IFJ [34]. During the flexible switching through decision making, the IFJ initially leads to the recruitment of related L‐FPN and M‐CIN networks in a functional connection manner [35]. Dynamic variations in network topography and connectivity in flexibility‐related brain regions lead to personalized differences in task performance. Other results confirm that increased activity of dlPFC is associated with CF and switching demands in children and adults [36, 37, 38].

Local atrophy in subcortical nodes like the caudate nucleus is associated with deficits in CF and switching capacity, coinciding with significant cognitive load and error rate during task‐switching [39]. Similarly, traumatic brain injury (TBI) alters white matter microstructure and disrupts upper radiation from the caudate nucleus to the auxiliary motor zone, which significantly correlates with the switching load and impaired CF [40].

Animal models further verify the neural basis of CF. In marmoset monkeys, the lesion of the lateral PFC disturbs extradimensional shifts without any perturbation in reversal learning or switching responses to the previously rewarded alternative, whereas the orbitofrontal cortex (OFC) lesion has the opposite effect. These results demonstrate a functional dissociation in which the lateral PFC supports shifting between abstract perceptual dimensions, while the OFC and related circuits participate in responses to reinforcement‐based changes [41]. Similar patterns have been verified in rodent models, such that OFC lesion impairs reversal learning due to the perseveration of previous selections [42]. The dorsomedial striatum lesions disturb both shifting and reversal tasks, affecting the ability to update behavior and inhibit habitual responses. This region interacts with multiple PFC regions to create new strategies, supporting flexible behavioral adaptation [43]. Overall, the acquisition of CF is a process that happens with the participation of different brain parts during development from childhood to adulthood. In the next section, the development process of CF will be discussed.

## 4 Development of CF

CF follows an inverted U‐shaped trajectory through the life span, reaching its peak in the second decade of life and declining in later decades [44]. While the foundational forms of executive functions are observable in the first year of life, most studies on CF have been done in preschool and primary school periods to evaluate the development of its components. However, changes in these components are also evident throughout adulthood [45]. As investigated by neuroimaging, behavioral, and clinical studies, CF development is driven by both neural circuits' maturation and response to environmental demands. Numerous studies have highlighted the participation of various brain regions in CF, including the left parietal cortex, right inferior PFC, striatum, and anterior cingulate cortex (ACC) [46, 47]. CF development depends on inhibitory control, working memory, language ability, emotional understanding of verbal communication, and Theory of Mind (TOM) skills during preschool, such that these capacities interact and lead to flexible cognitive behaviors in preschoolers [48]. Perseveration, which refers to the tendency to persist with an outdated mental framework in response to updated environmental stimuli, is a hallmark of immature CF and typically decreases with age [49]. In a study on preschoolers, the Dimensional Change Card Sort task (DCCS) was used to test CF. The children were asked to sort the two‐dimensional cards in one dimension (e.g., color) and then to rearrange the cards in another dimension (e.g., shape). While 4‐year‐olds could rearrange the past dimension to a new dimension, most 3‐year‐olds preserved the previous dimension [50]. These age‐dependent differences confirm CF‐related capability over time [51].

The DCCS studies demonstrate that switching ability in 3‐year‐olds correlates with right inferior PFC activity, while in 5‐year‐olds, it is associated with bilateral PFC activation, showing increased efficiency in neural processing [52, 53]. Interestingly, different subregions of the PFC mature at varying rates. For example, the OFC tends to mature earlier than the dlPFC, which participates in distinct functional processes during development [54]. Also, the maturation of these subregions is directly related to the enriched environment that contributes to synaptogenesis development and the creation of new neural circuits in the medial PFC, perirhinal cortex, and OFC that consequently enhance CF performance [55, 56]. The CF can be affected by various neurocognitive disorders, which will be discussed in the next section.

## 5 The Effect of Neuropsychiatric Conditions and Context on CF

CF is a complex hierarchical process that can be influenced by neuropsychiatric conditions, especially under stressful situations [57]. The researchers reported that acute stress induction impairs CF in men more than women, as evidenced by increased perseverative errors—a prominent indicator of cognitive rigidity [58]. This vulnerability may be driven by gender and sex differences in the noradrenergic system, dopaminergic system, and expression level of the mu‐opioid receptor [57, 59, 60, 61, 62]. Also, this phenomenon positively correlates with psychopathological traits, like rumination [63] and suicidal behaviors [64]. Moreover, depending on its duration and intensity, anxiety impedes the ability to shift flexibly between strategies in response to changes in task demands and maintain a plan in the presence of distractors. The nature of these disrupted cognitive processes is consistent with dysfunctional PFC activity during anxiety [65].

Furthermore, CF deficits have been reported in several neurodevelopmental disorders, such as schizophrenia [66], children with attention deficit and hyperactivity disorder (ADHD) [67], and ASD [68]. Individuals with ASD have significant deficits in social communication and restricted and repetitive behaviors that are related to CF deficits in these patients [69]. Children with high‐functioning ASD demonstrate abnormal frontal theta oscillations at the late stage but not the early stage of the modified Wisconsin Card Sorting Test (WCST) task, supporting the presence of impaired CF in this population [70]. Boshomane et al. found no significant effect of sex and age on working memory and switching tasks in children with ADHD, though a deficiency in these functions was significantly correlated with academic and social performance [71]. Also, CF, as a core executive function, has been investigated in several studies on schizophrenia patients [72, 73, 74]. Klaus et al. found that monocyte chemoattractant protein‐1 (MCP1) chemokine is correlated with CF in schizophrenia, and patients with high MCP1 levels display increased switching errors during color–word interference tasks [73]. The following section discusses the specific questionnaires and tasks related to CF.

## 6 Measuring CF

CF can be assessed using a range of standardized questionnaires and cognitive tasks. Also, different components of CF can be identified through cognitive tasks combined with EEG recordings. The following sections introduce key questionnaires and tasks used to assess CF.

### 6.1 CF Scales and Questionnaires

#### 6.1.1 Cognitive Flexibility Scale (CFS)

The CFS is a well‐known psychological assessment tool developed to measure CF [75]. The CFS includes structured and valid questions that assess the focus shifting, task switching, and adaptive response across conditions [76]. This scale provides effective perspectives into problem‐solving skills, creativity, adaptability, and updated responses in dynamic contexts [77].

#### 6.1.2 Cognitive Flexibility Inventory (CFI)

The CFI is a brief self‐report scale designed to evaluate the CF necessary for challenging, problem‐solving, and updating thoughts with balanced and adaptive mental patterns [78]. The CFI evaluates three key components of CF. First is the analysis of a complicated situation and providing solutions via controlled cognitive shifts. Second, it evaluates the ability to present multiple explanations for events and behaviors, reflecting mental proficiency in considering various standpoints. Finally, the scale evaluates the capacity to find various solutions to complex problems, illuminating cognitive potential in generating adaptive behaviors [79]. Considering theoretical foundations, the CFI presents itself as a valuable tool in the assessment of CF complications, advancing our comprehension of its pivotal role in facilitating adaptive psychological functioning [80].

#### 6.1.3 Cognitive Control and Flexibility Questionnaire (CCFQ)

The CCFQ is a valuable tool in cognitive psychology, specifically designed to quantitatively evaluate the intricate interplay between cognitive control and CF, two interconnected cognitive processes that underlie adaptive functions and behavioral responses [7]. This psychometric assessment tool evaluates the complex dimensions of cognitive function, including attention regulation, suppressing irrelevant inputs, and smoothly shifting between tasks [81]. Additionally, the CCFQ is a well‐designed and validated tool, providing a comprehensive exploration of cognitive control and flexibility. Its inventory covers a wide spectrum, including the proficiency in maintaining focus, inhibiting impulsive reactions, and creating adaptive approaches when confronted with challenges [82].

### 6.2 CF Main Tasks

CF tasks provide a tool, enabling the exploration of mechanisms underlying cognitive processes and behavioral responses related to switching strategies. These tasks evaluate an individual's ability to shift between mental frameworks in response to dynamic environmental inputs [31]. CF tasks cover diverse paradigms; among these, set‐shifting and task‐switching paradigms are the most prominent [83].

#### 6.2.1 Attentional Set Shifting

Attentional set‐shifting refers to the ability to shift between cognitive frameworks or task demands in response to dynamic environmental cues [84]. Typically, participants are presented with stimuli to switch their mental focus. The success in attentional set‐shifting orders involves ignoring previous cognitive rules and accommodating a novel framework that adapts to the novel stimulus parameters [85].

#### 6.2.2 Task Switching Behavioral Paradigms

Task Switching Behavioral Paradigms is a widely used tool for exploring the underlying mechanisms of CF and related adaptive processes [31]. These paradigms offer a systematic approach to evaluate shifting strategies between diverse cognitive tasks, effectively simulating real‐world scenarios necessitating cognitive adjustments [86]. Analyzing response times, error rates, and neural activations during the switch provides insights into the complexity of task preparation, the inhibition of external information, and cognitive control [87]. Overall, task‐switching paradigms shed light on the inherent neural circuitry and cognitive strategies accompanying adaptive task shifting, increasing our comprehension of executive functions and CF [88].

#### 6.2.3 Reversal Learning

Reversal learning is a widely used paradigm within cognitive research to evaluate dynamic cognitive processes that underlie CF and decision‐making [89]. This paradigm presents a well‐structured framework that evaluates an individual's capacity in response to changes in reward‐based contingencies [90]. In reversal learning tasks, participants initially learn how to associate specific responses with favorable consequences [91]. These associations are reversed in a particular situation, meaning that previously successful responses are ineffective. The success in reversal learning tasks depends on the capability to recognize and promptly adjust to these shifts [42]. By analyzing participants' response data, reaction times, and neural activations, researchers gain comprehensive insights into the complex cognitive mechanisms entailing the recognition and merging of novel information, the inhibition of outdated responses, and the behavioral adaptation strategies [92].

### 6.3 Selecting the Standard Measurement Protocol of CF

From the psychometric perspective, measuring CF is complicated by a well‐known problem named “task impurity” as a component of EF [93]. Considering this issue, CF tasks involve different loadings on its three prime components, including attentional load, working memory load, and a degree of response inhibition [94]. To reduce the “task impurity” issue and effectively measure the psychological construct of CF, evaluation can be executed by administering multiple CF tests that load on the component of interest and employing a latent variable approach to analysis [95]. Also, different dimensions of cognition are affected in various disorders, so depending on the purpose of the study, special attention should be paid to measuring desired indicators and components in the CF task analysis [96]. For example, commission errors, a type of error where the person presses a button twice instead of once, may be more common in obsessive‐compulsive disorder (OCD); it would be better to pay more attention to this index during the evaluation and analysis [97]. Also, using tools such as functional Near‐InfraRed Spectroscopic (fNIRs) or fMRI, which simultaneously show the activity of different parts of the brain by measuring HbO, HbR, and blood flow to the PFC while performing CF‐related tasks, can be very helpful in the correct and precise evaluation process [98, 99].

In the next section, noninvasive interventions that improve cognitive function will be discussed in detail.

## 7 The Role of Noninvasive Interventions in Improving CF

Noninvasive interventions, such as tDCS, rTMS, PBM, NF, and VR techniques, have been verified as an effective method to enhance CF (Tables 1 and 2). These methods have been established within research and clinical conditions as a potential approach to modulate neural activity and cognitive functions that underlie the foundation of CF [143]. While additional validation is necessary, these noninvasive interventions have shown promising effects for enhancing CF across clinical conditions and for promoting cognitive processes in healthy persons [144]. In certain techniques, such as tDCS, rTMS, and PBM, the neuromodulatory stimulation can directly induce biophysical changes at the neuronal and circuit levels in different brain regions [145]. The neuromodulatory change may also be achieved during the cognitive interaction and task performance, as in NF and VR techniques. The following section categorizes these neuromodulatory methods based on their mechanism of stimulation—direct or indirect approaches.

**TABLE 1:** Comparison of noninvasive neuromodulatory techniques in improving cognitive flexibility (CF).

Technique	Modality	Mechanism of action	Effects on CF
transcranial Direct Current Stimulation (tDCS)	Noninvasive stimulation by direct electrical current	Modulates cortical excitability by altering the membrane potential	Promising improvements: studies show enhanced CF, especially in prefrontal tasks, but some show no effect
repetitive Transcranial Magnetic Stimulation (rTMS)	Magnetic stimulation via repetitive pulses	Induction of neuroplastic changes by modulating synaptic plasticity and membrane potential	Positive effects on CF, particularly with DLPFC targeting, but some interventions show no effect
Photobiomodulation (PBM)	Low‐level near‐infrared light therapy	Improves mitochondrial function and cortical blood flow	Positive effects on CF, particularly in neurological conditions with cognitive deficiencies
Virtual reality (VR)	Computer‐simulated interactive and dynamic environments	Provides immersive nature and ecologically valid cognitive tasks	Effective in improving CF via real‐life simulation
Neurofeedback (NF)	Real‐time EEG‐based self‐regulation	Voluntary modulation of brainwave activity	Generally positive effects on CF, especially in ADHD and ASD children

**TABLE 2:** The role of noninvasive neuromodulatory techniques in improving cognitive flexibility (CF).

Intervention	Sample	Application method	Study design	Key results of CF	Effect duration	References
tDCS	Healthy adults	dlPFC (2 mA)	Single session, within‐subject	Improvement in Stroop and Stop Signal performance during active stimulation	Not studied	[100]
tDCS	Healthy students (*n* = 43)	Left dlPFC (HD‐tDCS)	9 sessions, Single‐blind, with fNIRS	Improvement in SAT indices Reduced HbO in dlPFC linked to Stroop	Observed after 9 sessions; no long‐term follow‐up	[101]
tDCS	Healthy students (*n* = 36)	Left dlPFC (1.5 mA)	Double‐blind, sham‐controlled, with APTD	Reduced Perseverative errors in WCST Dopamine increases in reaction time tDCS + APTD restored performance to control levels	Only immediate effects postintervention were studied; no long‐term follow‐up	[102]
tDCS	Parkinson's patients (*n* = 18)	Left & Right dlPFC (2 mA)	Double‐blind, sham‐controlled, 10 sessions	Improvement in Trail Making Test B performance in both left and right dlPFC stimulation groups	Improvement sustained up to 1 month postintervention	[103]
tDCS	Healthy adults (*n* = 60; 30 males, 30 females)	mPFC (1.5 mA)	Randomized, double‐blind, sham‐controlled	Increased reaction time in the theory of Mind task in females; no effect in males or cathodal stimulation	Only immediate effects postintervention were studied; no long‐term follow‐up.	[104, 105]
tDCS (Anodal tDCS)	Parkinson's patients with mild cognitive impairment (PD‐MCI) (*n* = 20)	Medial Frontal Cortex (MFC) (1.5 mA)	Randomized, double‐blind, sham‐controlled	Reduced reaction time in the theory of mind tasks: no effect on accuracy	Only immediate effects postintervention were studied	[104, 105]
tDCS	Mild Alzheimer's patients (*n* = 30; mean age 75.6)	Left dlPFC (2 mA)	Randomized, double‐blind, sham‐controlled, cross‐over	Improvement in CASI, MMSE, WCST, especially in concentration, orientation, language, verbal fluency, concept formation, and abstract thinking	Only immediate effects postintervention were studied; no long‐term follow‐up	[106]
tDCS	Gambling disorder patients (*n* = 20)	Bilateral dlPFC (Right Anodal/Left Cathodal, 2 mA)	Triple‐blind, sham‐controlled, parallel design, three sessions (every 2 days)	Improvement in decision‐making (Iowa Gambling Task) and WCST indices compared to sham	Only immediate effects postintervention were studied; no long‐term follow‐up	[107]
tDCS	Men with opioid use disorder under methadone therapy (*n* = 31)	Bilateral dlPFC (2 mA)	Double‐blind, sham‐controlled, pretest/posttest	Improvement in Planning, decision‐making, inhibitory control/selective attention, and memory	Only immediate effects postintervention were studied; no long‐term follow‐up	[108]
tDCS	Children with ADHD (*n* = 25)	Left dlPFC (Anodal)/Right dlPFC (Cathodal)	Double‐blind, sham‐controlled, pretest/posttest	Improvement in inhibitory control (Go/No‐Go), working memory (N‐back), switching (WCST), and interference control (Stroop)	Only immediate effects postintervention were studied; no long‐term follow‐up	[83]
tDCS	Teens and adults with ASD (*n* = 41)	Left dlPFC (Cathodal)/Right Supraorbital (Anodal)	Double‐blind, randomized, sham‐controlled, 2 weeks	Improvement in social performance, emotion recognition, shifting, and processing efficiency correlated with enhanced functional connectivity in the right prefrontal cortex	Only immediate effects postintervention were studied; no long‐term follow‐up	[109]
tDCS	Adults with ASD and dysexecutive syndrome (*n* = 10)	Right supraorbital (Anodal)/Left dlPFC (Cathodal)	Open‐label, before‐and‐after, 10 sessions, 2 mA, 15 min	Improvement in TMT‐A/B initiation time Reduced repetitive behaviors (RRB) and BDSI scores	Effects sustained at 3‐week follow‐up	[110]
rTMS	Women with Major Depressive Disorder (*n* = 28)	Left dlPFC (20 Hz)	Double‐blind, sham‐controlled, 10 sessions	Reduced depression severity (BDI), improved accuracy, and Reaction time in Go/NoGo and perseverative and nonperseverative errors, and Failure to Maintain Set in WCST	Effects sustained at 1‐month follow‐up	[111]
rTMS & TBS	Suicide attempters (*n* = 30)	The prefrontal cortex (1 Hz)	Double‐blind, sham‐controlled, 10 sessions	Improvement in selective attention, working memory, switching, and reaction time	Effects sustained at 1‐month follow‐up	[112]
rTMS	Young adults with depression (*n* = 50)	Left dlPFC (10 Hz)	Randomized, sham‐controlled, 20 sessions	Improvement in depression symptoms and switching	Effects sustained at 1‐month follow‐up	[113, 114]
rTMS	Traumatic brain injury patients (*n* = 20)	Left dlPFC (1 Hz)	Randomized, controlled trial	Improvement in depression and switching	Effects sustained at 1‐month follow‐up	[115]
iTBS	Adults with Autism (*n* = 20)	Bilateral posterior superior temporal sulci	Pilot study, five sessions	Improvement in CF and social cognition	No follow‐up study	[116]
Accelerated rTMS (5 Hz, 3 sessions/day)	Healthy adults	Left dlPFC	Randomized, sham‐controlled, 5 days	Improvement in sustained attention and; decreased reaction time and increased response accuracy in attention‐switching tasks	Immediate effects; no long‐term follow‐up	[117]
PBM	Older adults (*n* = 48)	Frontal cortex (NIR light)	Randomized, double‐blind, sham‐controlled	Improvement in cognitive function, especially executive functions (verbal fluency, working memory, switching)	Immediate effects postintervention	[118]
						
PBM	Children with ASD (*n* = 10)	Prefrontal cortex (NIR light)	Retrospective study	Improvement in social functioning, communication skills, switching and attention.	Immediate effects postintervention	[119]
PBM	TBI patients (*n* = 30)	Frontal cortex, parietal cortex (NIR light)	Randomized, double‐blind, sham‐controlled	Improvement in cognitive rehabilitation includes improvement in executive function, CF, and processing speed.	Immediate effects postintervention	[120]
PBM	Older bipolar patients (*n* = 15)	Frontal cortex (NIR light)	Proof of concept study	Improvement in cognitive functions, especially memory, attention switching, and executive function	Immediate effects postintervention	[121]
PBM	Dementia patients (*n* = 24)	Prefrontal cortex, parietal cortex (NIR light)	Randomized, double‐blind, sham‐controlled	Improvement in cognitive function, including memory, switching and executive function (MMSE, Cognitive Function Test)	Immediate effects postintervention	[122]
PBM	Older adults with nonamnestic MCI (*n* = 8)	Frontal lobe (NIR light)	Case studies	Improvement in cognitive functions, CF and mental health outcomes (anxiety, depression)	Immediate effects postintervention	[123]
PBM	Chronic TBI patients (*n* = 14)	The prefrontal cortex, temporal cortex (red/NIR light)	Open‐protocol study	Improvement in cognitive performance, especially switching attention, processing speed, and memory	Immediate effects postintervention	[124]
VR (STRIVE system)	Military personnel predeployment	VR‐based emotional resilience training environment	Pilot and feasibility study	RIVE VR system showed promise in improving emotional regulation and resilience, with implications for enhancing cognitive flexibility under stress	Short‐term training effects; stress response assessed	[125]
VR	Individuals with Autism Spectrum Disorders	VR‐based simulated social environments	Review and exploratory trials	VR environments showed promise for improving social understanding and possibly enhancing cognitive flexibility in individuals with autism spectrum disorders	Not specifically quantified; described as a potential benefit	[126]
VR	Children with Autism Spectrum Disorder	VR cognitive training tasks	Experimental study	VR‐based cognitive rehabilitation improved contextual processing, with implications for enhancing cognitive flexibility in children with autism	Short‐term improvement postintervention	[127]
VR + Aerobic Exercise	Healthy young males	VR combined with aerobic training	Randomized controlled trial	Aerobic exercise combined with VR significantly improved cognitive flexibility and selective attention in young males	Short‐term effects postintervention	[128]
VR	Older adults with Mild Cognitive Impairment (MCI)	VR‐based physical and cognitive training environment	Randomized controlled trial	Combined VR physical and cognitive training significantly improved cognitive abilities, including cognitive flexibility, in elders with MCI	Short‐ to medium‐term effects observed postintervention	[129]
VR	Community‐dwelling older adults	Virtual cognitive training environment	Randomized controlled trial	VR‐based cognitive training significantly improved executive functions, including cognitive flexibility in older adults	Short‐term effects observed postintervention	[130]
VR	Healthy adults	VR‐based diversifying environment	Experimental study	Exposure to diversifying experiences in virtual environments significantly enhanced participants' cognitive flexibility	Immediately, postexposure	[131]
VR	Healthy adults	VR with simulated hallucinations	Experimental study	Simulated visual hallucinations in VR led to a significant increase in cognitive flexibility in healthy participants	Immediate, short‐term effect	[132]
VR	Healthy adults	VR‐based restorative environment	Randomized controlled trial	VR restorative environments enhanced creativity and cognitive flexibility compared to control environments	Short‐term effects postexposure	[133]
VR	Inpatients with schizophrenia	Ecological virtual reality environment	Pilot study	Improved flexibility after VR‐based ecological cognitive remediation	Short‐term effects observed	[133]
VR serious game training	People diagnosed with schizophrenia	Virtual reality serious game	Randomized controlled trial	VR serious game training improved cognitive functions, particularly in flexibility, attention and memory, in schizophrenia patients	Short‐term effects	[134]
VR	Healthy individuals, Schizophrenia patients	Virtual environment	Cross‐sectional, observational	Cognitive flexibility was assessed using a VR‐based task. Schizophrenia patients showed poorer cognitive flexibility compared to healthy individuals	Not mentioned	[135]
NF	Patients with mild cognitive impairment (MCI)	EEG‐based	Preliminary pilot study	Improvement in overall cognitive functions, including attention and memory	Short‐term (pilot data)	[136]
NF	Children with ASD; primary caregivers' reports	EEG‐based	Qualitative exploratory study	Perceived improvements in emotional regulation, behavior, flexibility, and social engagement	Not specified (subjective)	[137]
NF	Adolescents with autism spectrum disorder	EEG‐based	Randomized controlled trial	Improved affective functioning; limited cognitive flexibility enhancement	Posttraining (short term)	[138]
NF	Children with Autism Spectrum Disorders (ASD)	EEG‐based	Experimental (Controlled)	Enhanced executive function, working memory, attention, and cognitive flexibility	Short to mid‐term	[139]
NF	Children with ADHD with/without epilepsy	EEG‐based	Experimental controlled study	Cognitive flexibility improved, more pronounced in the nonepileptic group	Posttreatment (short term)	[140]
NF	Adults with binge‐eating disorder	EEG‐based	Randomized controlled pilot	Reduced binge eating, improved cognitive control/self‐regulation	Postintervention	[141]
NF	Chronic stroke patients	EEG‐based	Controlled experimental study	Induced neuroplastic changes, enhanced working memory and attention‐switching processes	Short to mid‐term	[142]

### 7.1 Direct Stimulation Approaches (Biophysical Modulation)

Direct stimulation approaches refer to techniques like tDCS, rTMS, and PBM that deliver a specific physical energy, such as electrical, magnetic, or photonic stimulation, to a target brain region [146, 147, 148]. These approaches induce biophysical alterations in targeted brain regions, such as modulation of membrane potentials, synaptic plasticity, and neurotransmitter release [149, 150]. The tDCS delivers low‐intensity electrical currents by scalp electrodes that modulate cortical excitability, cognitive control, and behavioral adaptation [151]. The rTMS employs magnetic pulses to stimulate brain regions that are associated with executive functions, cognitive shift, and control [111]. The PBM, using photonic stimulation, has shown positive effects on cognitive function via modulating neural functions that underlie mental processes, including CF [121]. Collectively, these methods potentially enhance various aspects of executive function, including CF, in both healthy participants and subjects with neurological conditions. The following section provides an intricate explanation of these direct stimulation approaches on CF.

#### 7.1.1 Transcranial Direct Current Stimulation (tDCS)

##### 7.1.1.1 The Mechanism of tDCS Action

**FIGURE 2:**
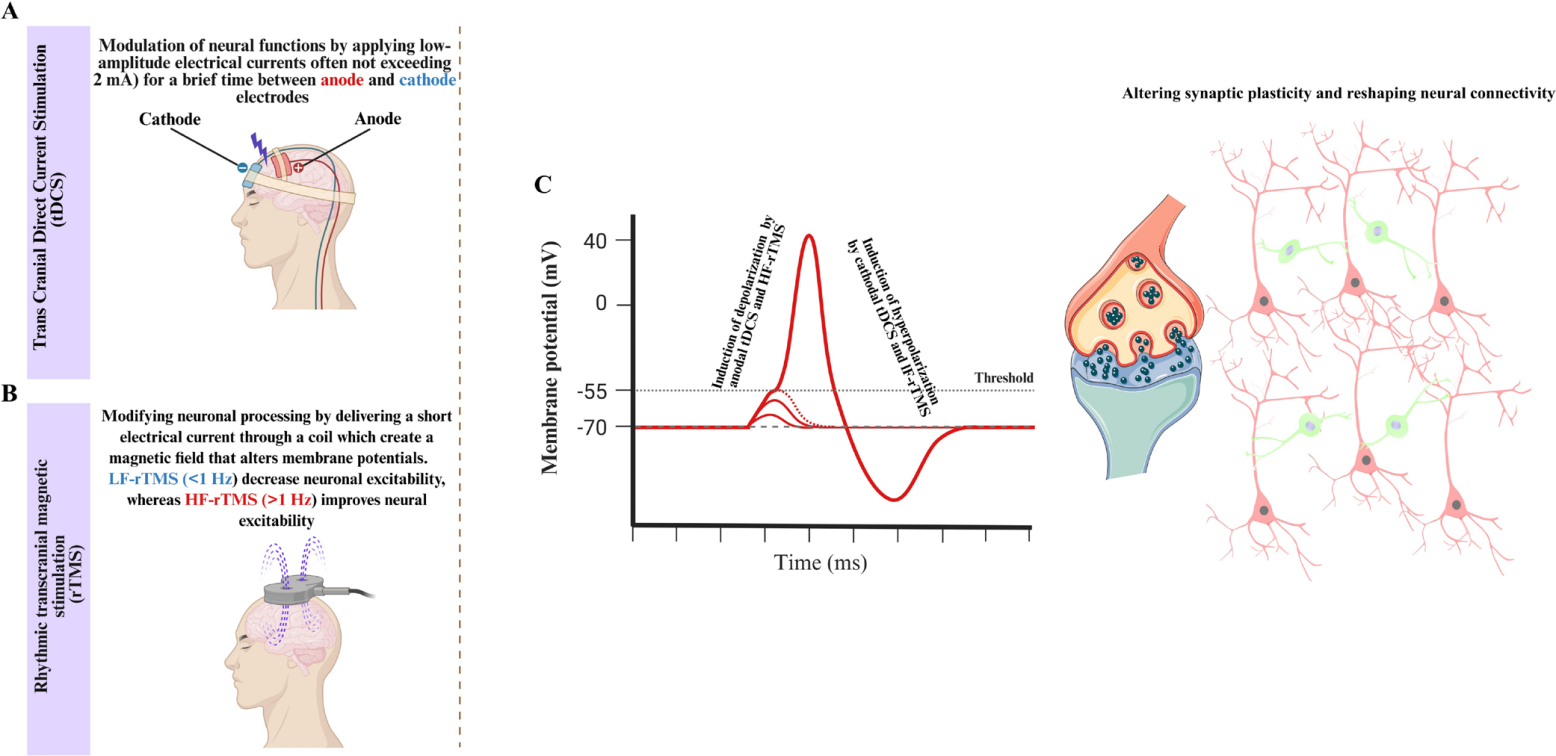
tDCS and rTMS mechanism of action. (A) tDCS is a noninvasive and safe neuromodulator technique that refines neural circuits via two electrodes that exert weak electrical current on specific sites of the brain over the scalp by low‐amplitude electrical currents (often not exceeding 2 mA) for a brief time (not surpassing 30 min) between electrodes (anode and cathode). (B) rTMS is a noninvasive and painless neuromodulator method that modifies neuronal processing of cortical regions by delivering a short electrical current through a coil, which creates a magnetic field that alters neural membrane potential. (C) Cathodal tDCS and low‐frequency rTMS (< 1 Hz) often decrease neuronal excitability and cause hyperpolarization of neurons; on the other hand, cathodal tDCS and high‐frequency rTMS (> 1 Hz) improve neural excitability and promote depolarization of neurons in cortical regions. These neuromodulator methods alter synaptic plasticity and reshape neural connectivity.

Transcranial direct current stimulation (tDCS) is a noninvasive and safe clinical neuromodulator method that refines cortical excitatory or inhibitory connections via two or more scalp electrodes that exert weak electrical current on specific brain regions [152]. Modulation of neural transduction is applied by low‐amplitude electrical currents (often not exceeding 2 mA) for a brief time (not exceeding 30 min) between anode and cathode electrodes [153]. Anodal stimulation increases the regional blood supply and promotes depolarization of neurons, while cathodal stimulation induces hyperpolarization, thereby inhibiting a specific brain region [154]. Through its modulatory effects, tDCS can reshape the neural connections in cognitive circuits—particularly those involving the dlPFC—thereby improving plasticity and cognitive function in many dimensions [155] (Figure 2A,C).

##### 7.1.1.2 tDCS Impact on Cognitive Function

Numerous studies have verified that tDCS can enhance cognitive functions in healthy subjects and clinical populations [156]. The mechanisms by which tDCS induces its neuromodulatory effects are long‐term potentiation (LTP) and long‐term depression (LTD), which are vital for plasticity, memory formation, and learning [157]. tDCS is a valuable neuromodulatory technique that can enhance learning and memory deficits linked to cognitive impairments in both healthy and neurological conditions [158]. Generally, this technique enhances motor function, learning, memory formation (including encoding, retrieval, and recognition of episodic memories), decision‐making, and switching strategies, all of which are related to CF [159, 160]. The following section declares the impact of tDCS on CF.

##### 7.1.1.3 Effects of tDCS on CF

**FIGURE 3:**
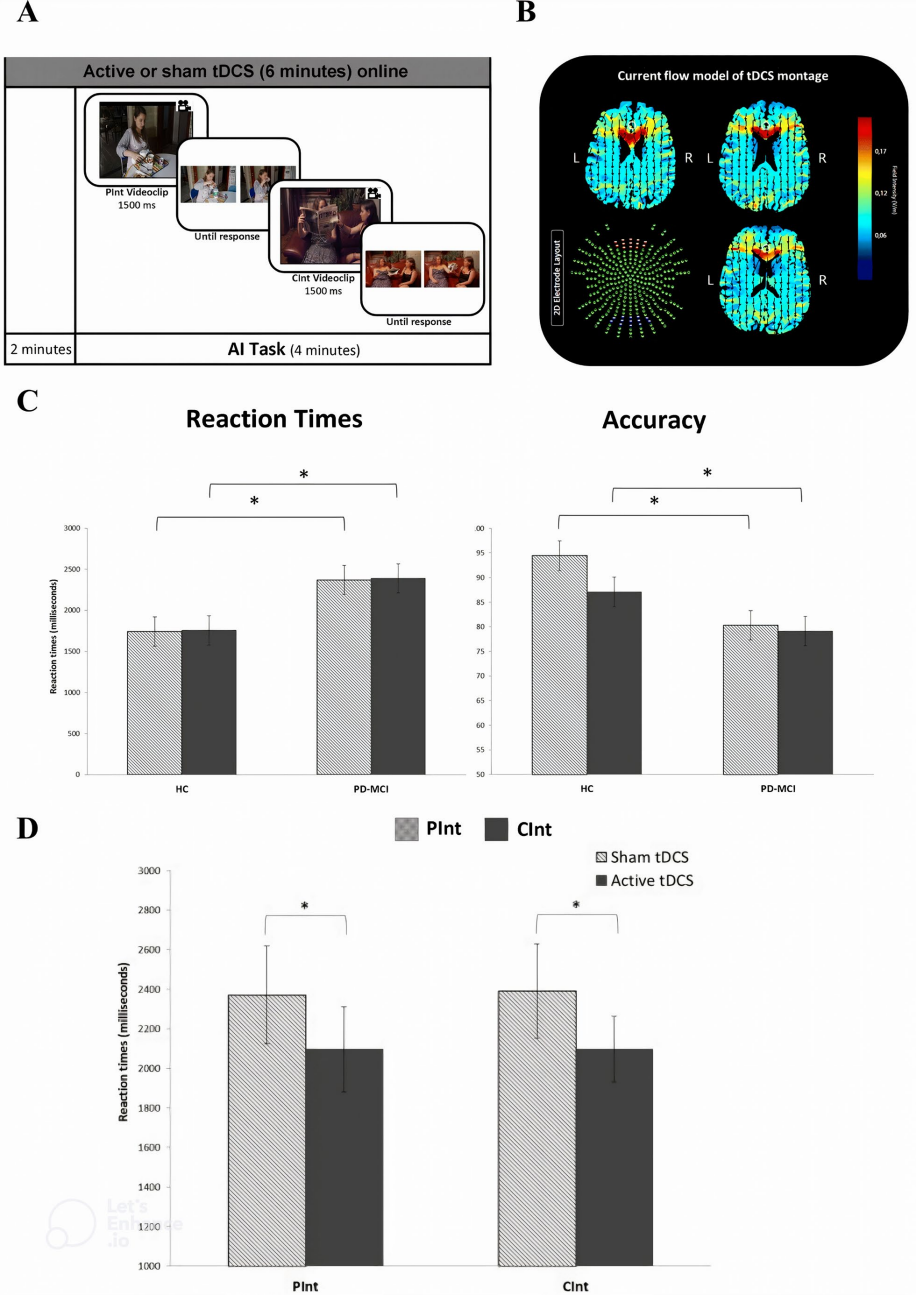
tDCS could ameliorate ToM deficits in PD. (A) The tDCS was applied 2 min before the onset of the experimental ToM block and extended until the end of the AI task, in which subjects were tasked with selecting a picture of the short video displayed, representing a logical story. The video ends with the push of one of the two buttons on the button box. CInt and Pint are examples of stimulus conditions. Private Intention condition (Pint): a condition in which subjects should identify another one's intent by watching isolated actions, such as hanging a picture on the wall. Communicative Intention condition (CInt): a condition in which subjects should recognize another one's communicative intent through social communication, like asking the other one to bring a glass of water for them. (B) The current flow model of tDCS is in which the anode electrode is placed on the medial frontal cortex, and the cathode electrode is located between Union and Oz. (C) The comparison of reaction times in the AI task through CInt and PInt conditions verifies that in patients with PD‐MCI, there is significantly worse accuracy and RTs in the CInt and PInt conditions compared to healthy controls. (D) The tDCS improves the RTs in PD‐MCI (*p < 0.05) [104, 105]. Reproduced with permission.

Evidence suggests that short‐term anodal tDCS over the dlPFC improves executive function proficiency, including CF [100]. In a study by Hongliang Lu and colleagues, three main components of executive functions (inhibitory control, working memory, and CF) after 9 sessions of HD‐tDCS (1.5 mA, left dlPFC, 20 min per session) in combination with fNIRS monitoring were improved in the anodal group as evaluated by the Shifting Attention Test [101]. Converging findings confirm that tDCS exerts its therapeutic effects by modulating dopaminergic neurotransmitters; for instance, stimulation of dlPFC increases dopamine neurotransmitter in the striatum [161, 162]. Cathodal tDCS improves working memory indices in a dose‐dependent manner [163], and when combined with tyrosine, a catecholaminergic precursor, it has the most synergistic effect on probabilistic reversal learning and task‐switching paradigms [164]. This finding provides some behavioral evidence supporting the modulatory effect of tDCS on dopaminergic tone and CF [165, 166]. Similarly, anodal dlPFC tDCS significantly reverses the switch failure during the WCST, caused by acute tyrosine/phenylalanine depletion and a drop in dopamine levels. This finding suggests that stimulation of the dlPFC can restore the adverse effects of decreased dopaminergic tone [102, 167]. Beyond a healthy population, tDCS has shown satisfactory effects in neurological or psychological conditions. Many pieces of evidence suggest the efficacy of tDCS either alone or in combination with complementary approaches like virtual reality‐based interventions, in enhancing motor learning, motor control, and cognitive function in poststroke recovery [168]. In a study by Liu and colleagues, combining tDCS with cognitive training in people with Parkinson's disease (PD) improved the gains in most indices of Mini‐Mental State Examination (MMSE), Montreal Cognitive Assessment (MoCA), Activities of Daily Living (ADLs), Stroop Color and Word Test (SCWT), and especially in CF‐related tasks including WCST and Digit Span Test (DST) [103]. Considering the dopaminergic system as a key factor in executive functions and CF, dysfunction of this system is related to some deficits in PD [169]. The tDCS could serve as a rehabilitation strategy to enhance cognitive function, executive function, working memory, and ultimately ADLs [170, 171]. The tDCS over the Medial Frontal Cortex (MFC) improves the characteristics of the ToM, a theory that is wrapped by CF and evaluated by the Reading Mind‐in‐the‐Eyes (RME) and Attribution of Intentions (AI) tasks [104, 105]. In patients with Parkinson Disease‐Mild Cognitive Impairment (PD‐MCI), tDCS over the MFC improves ToM deficits so that the reaction times of patients decrease in the AI task, though significantly remain higher compared to the healthy control (Figure 3) [105]. In Mild AD, applying tDCS over the left dlPFC and the right supraorbital cortex improves concentration, orientation, language ability, and WCST scores in all domains of “concept formation” “abstract thinking” “accuracy” and CF [106].

Cognitive inflexibility is a common manifestation of Major Depressive Disorder (MDD) reflected in difficulties with switching tasks and adapting to new conditions [172]. Depression may cooccur with disorders like a gambling disorder [173], and tDCS may be a promising neuromodulatory approach in this disorder. Alternate‐day sessions of right anodal/left cathodal tDCS over the dlPFC improve decision‐making and CF in participants with gambling disorders who executed the Iowa Gambling Test (IGT) and the WCST [107]. Also, bilateral dlPFC stimulation using tDCS in men with opioid use disorder receiving methadone therapy enhances memory, inhibitory control/selective attention, planning, decision‐making, and CF scores on the Cognitive Abilities Questionnaire (CAQ) [108].

Cognitive inflexibility is a core cognitive deficit in adolescents and young adults with neurodevelopmental disorders, and evidence suggests that tDCS can ameliorate this deficit [174, 175]. In ADHD subjects, administration of tDCS over the dlPFC and OFC improves CF and switching performance, as measured by WCST [83]. Also, repeated prefrontal tDCS sessions in ASD subjects significantly enhance their social cognitive processes and prefrontal resting‐state functional connectivity. These improvements were closely associated with emotional recognition enhancement and CF, especially in the “cold” executive function category, which was measured with the CANTAB Emotion Recognition Task (ERT), Color Trail Test—part 2 (CTT2), CANTAB MTT, and WCST [109]. In a study by Rothärmel, M. et al., subjects with high‐function ASD received 10 consecutive cathodal tDCS sessions (2 mA) over the left dlPFC for 15 min. Following intervention, there was a significant improvement in the initiation, hypoactivity, and especially CF that was evaluated by the Stroop test, Trail Making Test‐A and B (TMT A and B), and Modified WCST [110].

Despite promising findings, tDCS effects are highly variable due to stimulation modalities (reference/return electrode position) and biological heterogeneity [176]. For instance, a study found that tDCS with different locations, durations, and intensities of stimulation has no effect on cognitive performance criteria in patients suffering from ischemic stroke, while improving the hemi‐spatial neglect symptoms [177]. Research by Koshikawa and colleagues evaluated the effects of tDCS on CF in individuals with MDD. The anodal tDCS was exerted over either the dmPFC or the dlPFC, and WCST was executed before and after the tDCS application. The results showed that anodal tDCS over the dlPFC had no significant effect on improving CF, neither in participants with MDD nor in controls. Also, anodal tDCS over the dmPFC significantly reduced switching load, but only in participants with MDD [178]. Another study investigated the effects of tDCS on various executive functions in children with ADHD, including CF. Findings verified that stimulation of the dlPFC did not result in a significant CF improvement; however, applying tDCS simultaneously over the dlPFC and OFC significantly improved task‐switching performance [83]. A meta‐analysis on older subjects with cognitive impairment indicated enhancements in global cognitive function and selective attention after the tDCS intervention. This study identified a limited effect on CF, showing variable results among diverse populations and studies [179]. Overall, multiple factors contribute to the variability of tDCS outcomes. Anatomical variability can affect the efficacy of tDCS interventions, and individuals with lower baseline abilities will gain greater benefits from these interventions [180, 181]. Moreover, the tDCS appears more effective in tasks of moderate difficulty, whereas tasks that are excessively easy or overly challenging may not demonstrate consistent results from stimulation [182].

##### 7.1.1.4 The Potential Adverse Effects of tDCS

The tDCS is typically regarded as a safe, noninvasive neuromodulator method; however, some cases may report modest, transient adverse effects. The most reported adverse effects consist of itching, paresthesia, headache, burning sensation, and skin redness beneath the stimulation site [183]. Infrequent side effects include moderate weariness, concentration difficulties, dizziness, and nausea. Also, there have been occasional reports of seizures or extended hospitalization [184].

#### 7.1.2 Repetitive Transcranial Magnetic Stimulation (rTMS)

##### 7.1.2.1 The Mechanism of rTMS Action

rTMS is a noninvasive and safe neuromodulatory technique that modifies neuronal transduction of the cortex by applying a short electrical current through a coil, which creates a magnetic field that depolarizes neural membranes and induces action potentials [185]. The coil structure and design are critical factors in determining the depth and precision of stimulation. Low‐frequency rTMS (LF‐rTMS) (< 1 Hz) often decreases neuronal excitability, leading to inhibitory effects on the cortex. In contrast, high‐frequency rTMS (HF‐rTMS) (> 1 Hz) improves neural excitability, resulting in excitatory effects on cortical regions [186] (Figure 2B,C).

##### 7.1.2.2 rTMS Impact on Cognitive Function

rTMS could be employed to stimulate the cerebral cortex, offering a tool to investigate the mechanism of a specific brain region, like motor‐related functions and cognitive functions [187, 188]. rTMS effects have been extensively studied on the cognitive function of both healthy individuals and various neurological populations, including mild cognitive impairment (MCI), AD, and poststroke cognitive dysfunctions [189]. In a mechanistic study, the “AX” Continuous Performance Task (AX‐CPT) was administered after receiving HF‐rTMS to elucidate the dlPFC function in proactive and reactive control. Reaction times data illustrated that the left dlPFC is responsible for proactive control and the right dlPFC supports reactive control [190]. Application of rTMS to the left dlPFC improves global cognitive function, memory, language, and executive functions in individuals with AD and MCI [191]. Considering poststroke cognitive impairments, applying rTMS to the left dlPFC has been shown to improve global cognitive function, which was evaluated with the MoCA, Victoria Stroop Test, and Rivermead Behavior Memory Test [192]. The effects of rTMS on CF have also been investigated in various studies, which are discussed in the following section.

##### 7.1.2.3 Effects of rTMS on CF

Several studies have confirmed the beneficial effects of the HF‐rTMS over the PFC, showing improvement in various dimensions of cognitive functions, including working memory, executive function, attention, and CF, both in healthy subjects and those with neuropsychiatric disease [111, 193]. Theta burst stimulation (TBS) is a recent stimulation protocol that closely adjusts to neural firing patterns of the brain, demonstrating significant impacts on cognitive function [194]. Both HF‐rTMS and intermittent TBS (iTBS) promote working memory, executive function, and performance of N‐back tasks and the WCST, as valid scales in the determination of CF [117]. In subjects with MDD, after applying HF‐rTMS (20 Hz) over the left dlPFC (2500 pulses per session), the CF of participants was evaluated by Go/NoGo and WCS tests, pre and posttreatment. Findings indicated that rTMS significantly decreased the severity of depression and positively affected the perseverative/nonperseverative errors through WCST [111]. Also, a study by Rahimi et al. confirmed that rTMS and TBS significantly affect congruent reaction time, simple reaction time, and selective reaction time as assessed by SCWT, a test of CF [112]. In a similar study, depressed young adults received HF‐rTMS targeting the left dlPFC, and CF was assessed pre and posttreatment using TMT‐B. Results showed that HF‐rTMS treatment significantly correlated with verbal learning, depressive episodes, and CF improvements [113]. Administration of LF‐rTMS (1 Hz) to the right dlPFC in individuals with TBI demonstrates significant enhancement in cognitive tasks evaluating flexibility and inhibitory control, including the TMT and the SCWT [115]. In a study, iTBS was applied over the posterior superior temporal sulcus in participants with ASD. The CF was measured with the WCST, and other behavioral changes were evaluated by the Autism Spectrum Quotient (AQ). The analysis verified that iTBS's impact on CF was affected by baseline cognitive and social‐communicative performance [116].

Additionally, rTMS treatment in individuals with insomnia led to a significant reduction in Insomnia Severity Index (ISI) and Pittsburgh Sleep Quality Index (PSQI) scores, along with a notable increase in CFI scores. The Number‐Letter (N‐L) task results demonstrated that the switching cost in reaction time and accuracy was significantly greater in the sham group compared to the intervention group. Furthermore, ERP analysis revealed that, under switch conditions, the amplitude of P2 in the frontal region was significantly greater in the intervention group than in the sham group. Simultaneously, the beta‐band Event‐Related Desynchronization (ERD) in the parietal region was significantly lower in the active group compared to the sham group [195].

**FIGURE 4:**
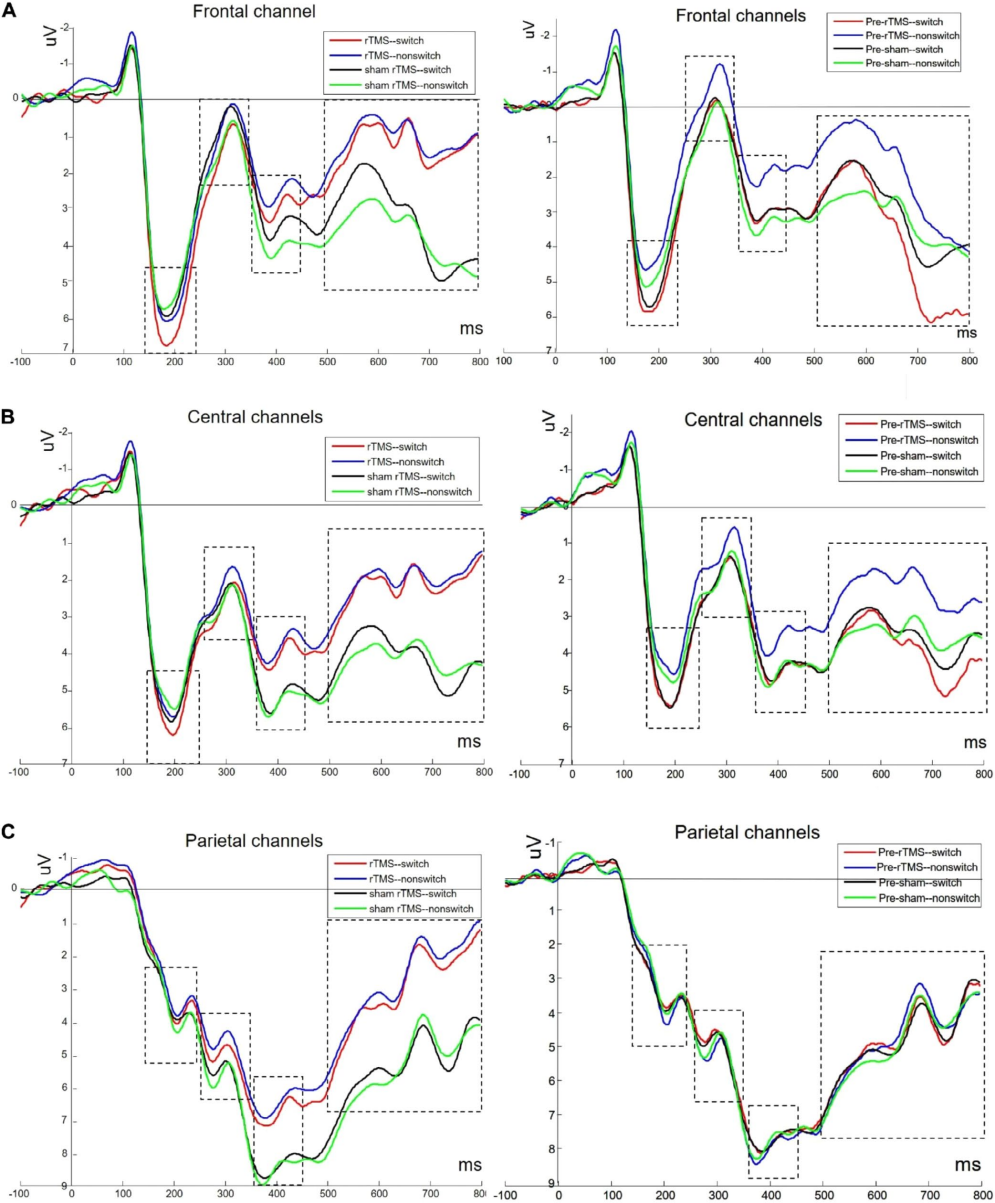
ERP components after the rTMS. Averaged ERP components (P2, P3, N2, LPC) in switch and nonswitch trials at frontal (A), central (B), and parietal (C) channels in the intervention (1 Hz rTMS) and sham (rTMS) groups through poststimulation and prestimulation conditions [196]. Reproduced with permission.

**FIGURE 5:**
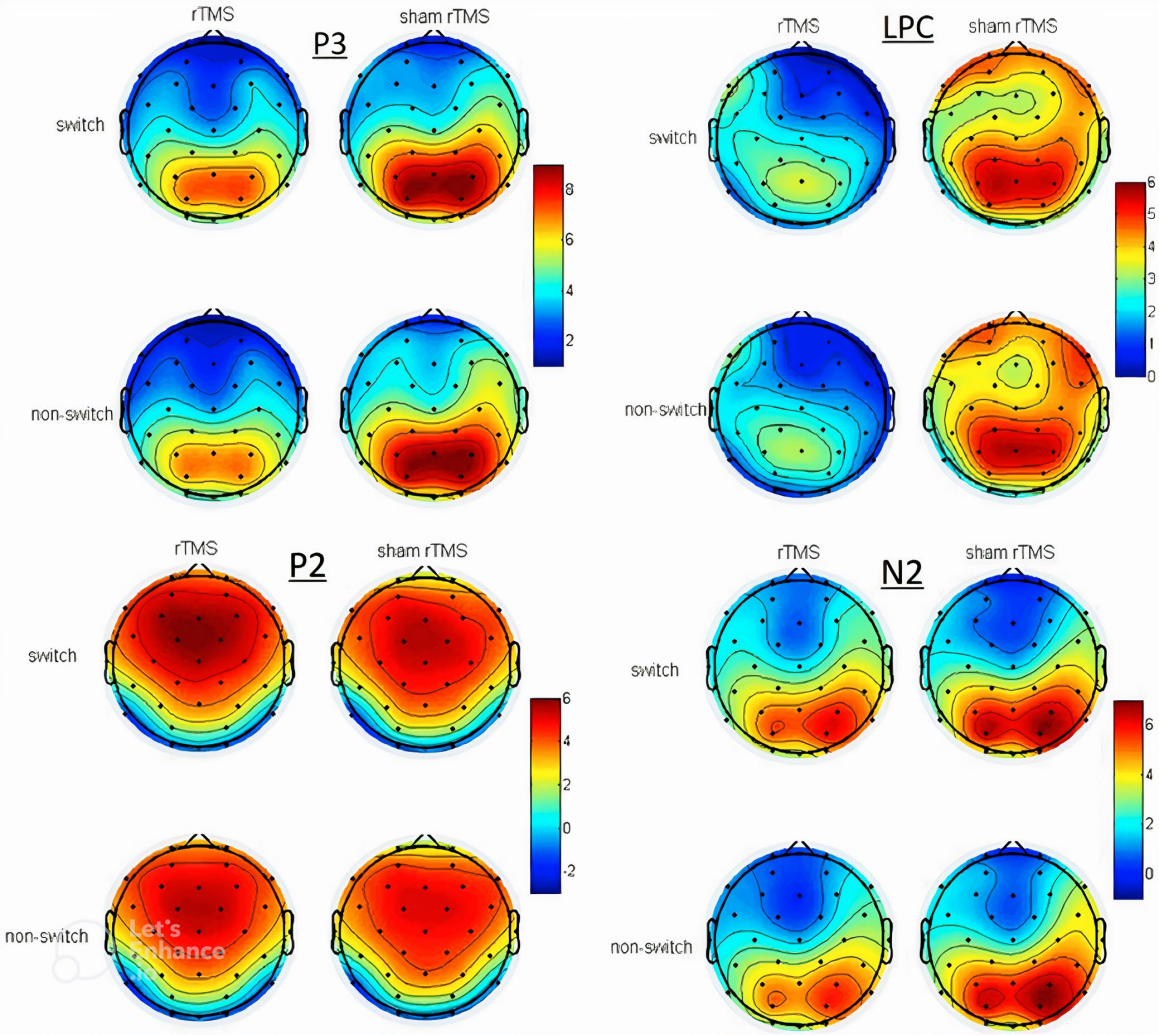
Topographic maps of ERP components after the rTMS. Topographic maps of ERP components (P2, P3, N2, LPC) voltage distribution in switch and nonswitch trials of intervention (1 Hz rTMS), and sham (rTMS) groups, through poststimulation and prestimulation conditions [196]. Reproduced with permission.

Given that various factors, such as brain network properties, can affect the responsiveness to rTMS, some inconsistencies have been reported about the rTMS effect on CF. For example, in a study by Liu and colleagues, the rTMS (1 Hz rTMS or sham rTMS) was applied to the dlPFC and had different effects on various components of ERP in different locations of the brain, such that it increased the P2 amplitude in switch trials but also led to increased N2 amplitude at nonswitch trials compared to the sham group. Also, rTMS significantly decreased the amplitude of P3 and late positive component (LPC) (Figures 4 and 5), demonstrating that targeting left dlPFC can reduce CF [196]. In another study, administration of 10‐Hz rTMS to the left dlPFC in schizophrenia patients showed no significant differences pre and postintervention in any CF tasks, including the Rey Auditory Verbal Learning Test, TMT A and B, WCST, DST, and the Regensburg Word Fluency Test, for both intervention and sham groups. Both groups demonstrated significant improvement over time; nevertheless, effect sizes suggest a numerical, albeit statistically insignificant, advantage of active rTMS in specific cognitive assessments [197]. In a similar study exploring rTMS effect on treatment‐resistant depression, subjects with suicidal thoughts/behaviors showed a significant improvement in emotional recognition and a considerable decrease in depressive symptoms and suicidal thoughts. However, there was no significant change in CF, which was evaluated by intra/extra Dimensional Set Shifting Test (IED) [198]. One of the main reasons for inconsistent and interindividual differences in response to various rTMS frequencies is the difference in cortical excitability; the same stimulation protocol has varying effects across individuals, emphasizing the complexity of predicting rTMS outcomes [199]. Individuals with stronger preexisting neural connectivity between cortical regions exhibit attenuated responses to iTBS, suggesting that the baseline cortical network can influence the outcome of rTMS [200]. Besides individual differences, the variation of protocol frequency, intensity, duration, and other parameters across studies creates heterogeneous results [201, 202].

##### 7.1.2.4 The Potential Adverse Effects of rTMS

rTMS is generally regarded as a safe and noninvasive neuromodulator method. However, some subjects may experience transient and mild adverse effects, including scalp discomfort, headache, and muscle twitching [114]. There are some infrequent and serious side effects, such as seizures and the development of hypomania [203].

#### 7.1.3 Photobiomodulation (PBM)

##### 7.1.3.1 The Mechanism of PBM Action

**FIGURE 6:**
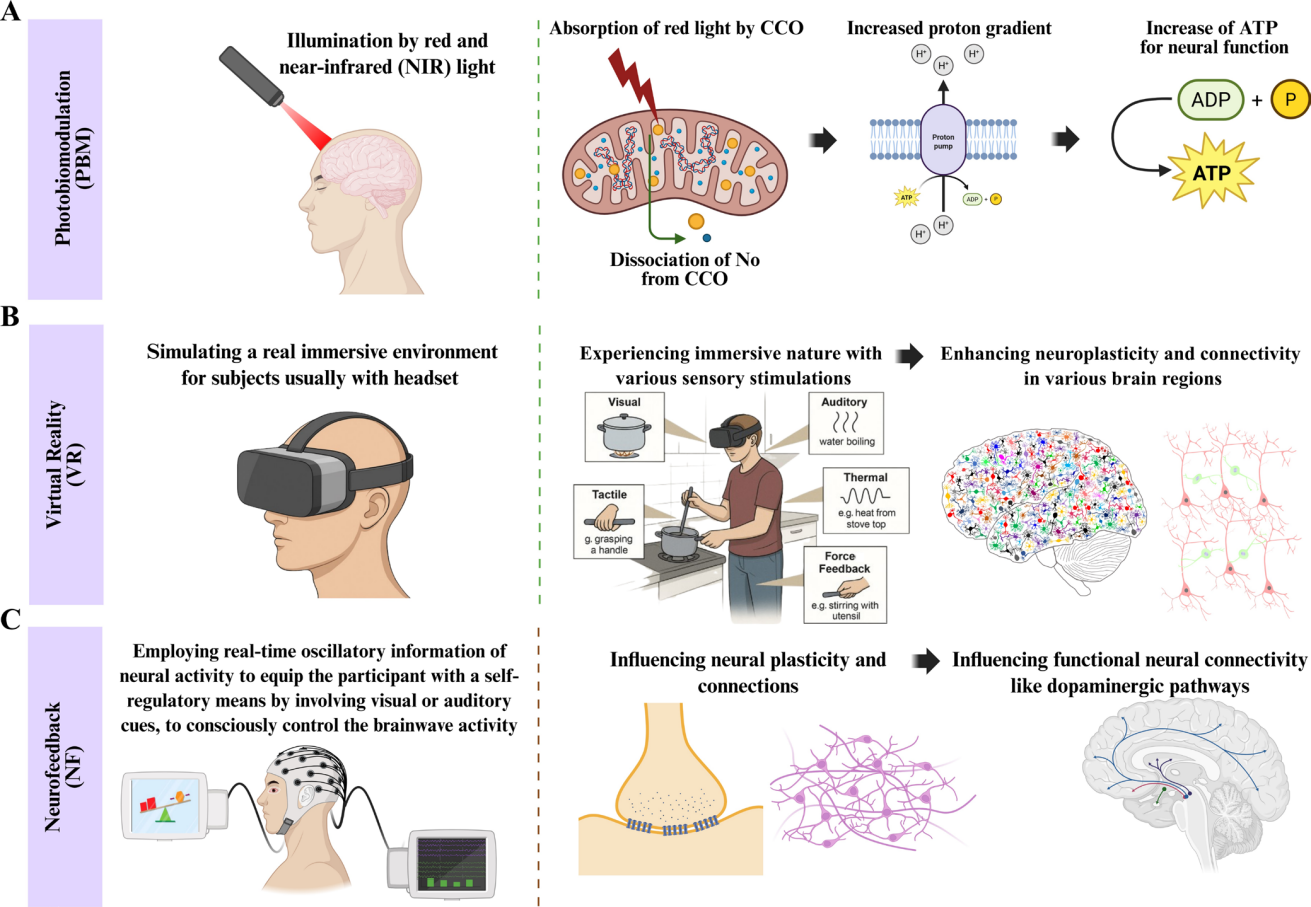
PBM, VR, and NF mechanism of action. (A) In PBM, following illumination of the skull by red and near‐infrared (NIR) light, mitochondrial CCO of neurons attracts the photons and triggers signaling pathways that lead to NO dissociation from CCO, proton pump activity enhancement, and oxidative phosphorylation in mitochondria. All of these lead to adenosine triphosphate synthesis and plenty of available energy for neural transduction. (B) VR, a phenomenal computer interface, simulates real‐time interaction via various sensory inputs. Immersion and interaction are two key features of VR that simulate a real immersive environment for subjects (like cooking in the kitchen) to respond in real time to motives by body movements naturally. All of these enhance neuroplasticity and connectivity in various brain regions. (C) NF employs real‐time oscillatory information of neural activity to equip the participant with a self‐regulatory means to modulate specific neural signals. It enables individuals to regulate their cognitive and emotional functions in reaction to online feedback that leads to the modulation of various neural circuits, such as dopaminergic pathways.

The PBM, initially termed “low‐level laser therapy,” leads to some immediate alterations in the metabolism of cellular organelles, especially mitochondria [204]. Upon exposure to red and near‐infrared (NIR) light, particular neural molecules attract the photons and trigger a change in metabolic reaction rate by activating specific signaling pathways [205]. Cytochrome c oxidase (CCO), the most abundant photo‐acceptor of red and near‐infrared light, is a critical enzyme in the mitochondrial respiratory chain [206, 207]. Absorption of photons by this acceptor leads to NO dissociation from CCO, which enhances proton pump activity and oxidative phosphorylation in mitochondria [205]. This cascade leads to the increased cerebral blood flow and adenosine triphosphate synthesis, thereby enhancing available energy for neural transduction [208, 209, 210]. PBM, as a noninvasive and developed interventional technique, has a promising effect on the cognitive function of human or nonhuman subjects [211] (Figure 6A).

##### 7.1.3.2 PBM Impact on Cognitive Function

The PBM‐induced metabolic alterations positively influence cognitive function, as evidenced by various human studies or animal models [212, 213]. PBM has also been investigated for its potential therapeutic potential in various neurological conditions, such as AD, aging, and TBI [214]. This noninvasive method enhances memory and executive functions in a population with AD, accompanied by a reduction in amyloid‐beta accumulation [215]. In older people, applying PBM on the forehead and assessing oxygenated hemoglobin (HbO) by fNIRS demonstrated that after PBM, participants showed significantly lower relative HbO during longer spans (i.e., span 7) of the visual span task. This may show less cognitive load when solving the difficult trials of the task [216]. A case report describing the application of PBM in an athlete with a history of concussions, experiencing headaches and concentration problems, demonstrated significant enhancements after administering home‐based PBM. After applying 8 weeks of PBM using LEDs that emitted 810 nm light pulsing at 10 or 40 Hz through an intranasal module and four transcranial modules, there was a substantial improvement in brain volume, functional neural connectivity, cerebral perfusion, and cognitive function [217]. There are several studies that have investigated the effect of PBM on CF, which are discussed in the following section.

##### 7.1.3.3 Effects of PBM on CF

The evidence supports the notion that PBM can improve CF and PFC functions safely and cost‐effectively. In healthy subjects employing light‐emitting diode clusters as a PBM intervention (633 and 870 nm, 1349 J) over both sides of the forehead and posterior midline significantly improved action selection, inhibition, and CF, which were evaluated by the Eriksen flanker and category fluency tests [118, 218]. In children with ASD, 6 months of home‐based transcranial PBM, using alpha and gamma protocols, significantly decreased cognitive and behavioral rigidity. This finding verifies an improvement in CF, as assessed by the Montefiore Einstein Rigidity Scale–Revised (MERS–R) [119]. Furthermore, the combination of PBM with mild hyperbaric therapy (mHBT) and molecular hydrogen therapy (MH) in individuals with mild to moderate TBI resulted in significant enhancements across all metrics of the TMT‐A and B. Considering ERP, there was a 28.3 ms decrease in P300 latency, a 1.2 mV increase in P300 voltage, and faster completion times for TMT‐A and B [120].

Transcranial Infrared Laser Stimulation (TILS), a safe and novel form of photobiomodulation, improves cognition by increasing prefrontal oxygenation and upregulation of the mitochondrial CCO [219]. Applying TILS to the bilateral PFC in older euthymic bipolar patients significantly improves the percentage of correct trials in the continuous performance task (CPT). Also, it hastens the completion time of TMT‐B, which evaluates visual attention and CF through task switching [121]. The treatment of dementia patients using low‐emission transcranial near‐infrared (tNIR) leads to enhancement of cognitive function, evidenced by increased scores in Logical Memory Tests I and II, the Boston Naming Test, and TMT A and B, which assess CF. Additionally, there was an improvement in both Auditory Verbal Learning Tests across all subtest categories, along with a reduction in overall performance time [122]. In case studies involving three older adults with nonamnestic MCI after receiving PBM over 9 weeks, there was a reduction in intrusion and perseveration errors during verbal memory and fluency assessments, confirming improvement in CF [123]. Red/near‐infrared light‐emitting diode application in subjects with mild TBI by placing LEDs bilaterally across the frontal, parietal, and temporal areas led to a significant improvement in the California Verbal Learning Test (CVLT)‐II, and multiple dimensions of executive function, like inhibition control and shifting (switching) inhibition that was evaluated by the Stroop test [124].

In the animal model of neglectful parenting, maternal separation as an early life stress during the first postnatal days impairs CF and increases the general energy metabolism [220]; however, PBM treatment (1064 nm, 30 mW, 60 cycles) restores the CCO metabolism to balanced levels and improves CF, such that treated animals prefer the new reinforced quadrant through the MWM test [118]. In another study, following PBM therapy, the brain's neural network was evaluated in the basal state and during the execution of a reversal task. Posttreatment evaluation verified a significant decrease in the CCO levels in the medial septum, striatum, entorhinal cortex, amygdala, thalamus, hippocampus, mammillary nuclei, and VTA [221]. Specifically, PBM therapy leads to CCO reduction in brain regions that participate in the reversal task, including CA1, CA3, the entorhinal cortex, and the accumbens. This posttreatment modulation of neural networks aligns with reversal memory improvement and preserved CF [222].

##### 7.1.3.4 The Potential Adverse Effects of PBM

While PBM is a safe and well‐tolerated neuromodulator method, it may occasionally cause minimal side effects such as headache and hyperactivity in autistic children [223]. Also, some clinical findings have reported moderate side effects like irritability, insomnia, “vivid color perception,” and “an ashtray‐like taste” [224].

### 7.2 Indirect Cognitive‐Interactive Approaches (Behavioral Modulation)

In contrast to direct stimulation approaches, indirect cognitive‐interactive approaches such as VR and NF modulate synaptic plasticity of neural circuits and shape behavioral responses by multisensory engagement during the performance of cognitive tasks. VR environments provide immersive and adaptable settings for practicing cognitive strategies in dynamic and interactive contexts that require problem solving, environmental adaptation, and task switching [121, 152]. NF technique offers a condition for individuals to interactively regulate their cerebral activity by providing real‐time monitoring and training of neural oscillations, thereby promoting the self‐regulation of cognitive states critical for adaptive function [111]. The subsequent sections elaborate on each of these indirect cognitive‐interactive approaches in intricate detail.

#### 7.2.1 Virtual Reality (VR)

##### 7.2.1.1 The Mechanism of VR Action

Virtual reality (VR) represents a cutting‐edge neurotechnology for enforcing neurorehabilitation. VR, a sophisticated computer interface, simulates real‐time motor and cognitive interaction via numerous sensory inputs [225]. Immersion and interaction are two key features of VR that simulate a real, engaging environment for subjects to respond in real‐time to motives by natural body movements (Figure 6B) [226]. Based on the level of immersion, VR can be divided into three types: low immersion, semiimmersion, and full immersion [227]. In the low‐immersion type, the subject interfaces with the virtual context using conventional tools like a monitor and keyboard. The semiimmersive type includes interactive devices such as balance platforms, haptic gloves, and motion trackers, providing a more complex condition. The fully immersive VR is an experience that offers a highly realistic and sophisticated view for subjects using a head‐mounted display that locates them in a surrounding screenland [228, 229]. These setups integrate multiple sensory modalities, like touch and smell stimulation, to promote subjects' deep immersive experience [230].

##### 7.2.1.2 Impact of VR on Cognitive Function

VR as a cognitive rehabilitation approach is a promising treatment method in neurological conditions like MCI and stroke to facilitate cognitive function by using various platforms and training modules at different stages of recovery [231]. VR can be recruited for the assessment or treatment of learning and memory deficits in patients with memory deficits, with the capacity for translation to real‐world situations [232]. VR‐based treatments are notably effective in improving executive functions, such as problem‐solving, planning, and decision‐making [233]. Through this treatment approach, individuals can immerse themselves in ecologically valid and real‐world scenarios that demand strategic thinking. The majority of studies engage participants in simulations wherein they are involved in ADLs [234]. These scenarios demonstrate beneficial effects in the rehabilitation and preservation of cognitive capacities in subjects with stroke, AD, and brain injuries [235, 236]. In the next part, the promising effects of virtual reality on CF will be discussed in detail.

##### 7.2.1.3 Effects of VR on CF

**FIGURE 7:**
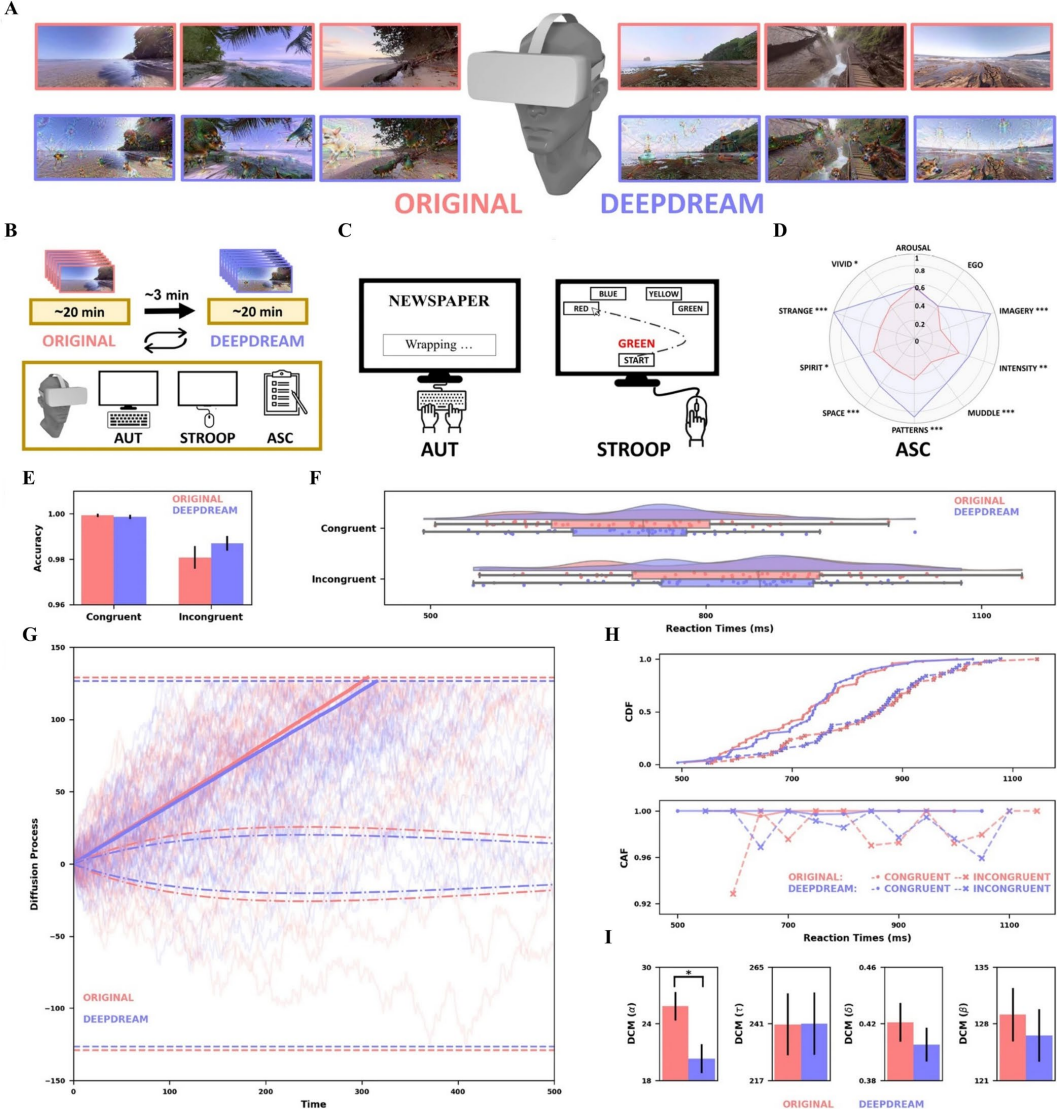
VR condition enhances cognitive flexibility. (A) VR conditions were panoramic 360° videos presenting natural scenes (red frames) and related Deep Dream counterparts (blue frames). (B) Conditions were presented across participants in a counterbalanced manner. (C) AUT and Stroop tasks followed VR conditions. (D) The Radar plot represents ASC results of OR (red) and DD (blue) conditions (Statistically significant, *p < 0.05; **p < 0.01, ***p < 0.001). (E) Accuracy bar plots in OR (red) and DD (blue) conditions through congruent and incongruent trials. (F) Raincloud plots of the Reaction times (RT) in the Stroop task through congruent and incongruent conditions. (G) Representation of results by Dynamic causal modeling (DCM). Semitransparent solid lines present simulated trials via resorted metrics from model fitting. Solid lines present the mean of simulated trials per condition. Dash‐dotted lines exhibit the autonomous procedure, while dashed lines demonstrate borders. (H) Both the cumulative distribution functions (CDF) and conditional accuracy functions (CAF) across stroop situations are fitted to the DCM. (I) Displaying the amplitude of the automatic process, the decay of the process, the drift of the controlled process, and the decision boundary range. All are indicated using the estimated parameters from DCM [196]. Reproduced with permission.

VR simulates real‐life environments that may evoke delusion‐like behaviors, such as paranoia, and subsequently facilitates the evaluation of cognitive domains like CF based on the adaptive responses in realistic conditions [237]. In one study, VR was used to measure the CF in immersive conditions, which required making rational decisions in real situations. The task showed reliable results in distinguishing the control population from schizophrenia individuals, such that schizophrenic subjects made maladaptive decisions that negatively correlated with the severity of negative symptoms [135]. Findings demonstrate that the VR‐based game training significantly enhances CF in individuals diagnosed with schizophrenia, evidenced by faster completion times on TMT‐A and B after the training program [134]. In a study, patients with schizophrenia underwent ecological virtual reality‐based cognitive remediation training (CR‐EVR), and incorporated tasks for enhancing inhibition, planning, working memory, self‐initiation, persistence, attention, and CF. The TMT was employed to assess processing speed, sequencing, CF, and visual‐motor skills, and results demonstrated a significant improvement in CF and other test domains following the intervention [133]. The VR improves CF to a greater extent, potentially exceeding even the effects of psychedelic drugs as a traditional CF modulator [238]. Following exposure of subjects to VR panoramic views and generating hallucinatory situations by the deep dream algorithm, their CF was measured via the alternative uses task (AUT) and Stroop task. Findings confirmed that altered perceptual conditions increased the flexibility of semantic networks, mirrored the mental effect of psychedelics, and enhanced the participation of automatic processes and chaotic dynamics of subjects. Therefore, CF was enhanced by simulated altered perceptual phenomenology, probably due to the reshaping of cognitive processing flow that hastens the detection of unusual strategies and inhibits automated usual choices [132] (Figure 7). According to investigations, creativity is inseparable from decisions in unexpected and extraordinary experiences, which require CF. These experiences involve “diversifying” aspects and active engagement, enhancing creativity and CF [239]. Another study examined a VR‐based cognitive intervention aimed at improving psychological processes, including planning, movement control, and adaptation, in community‐dwelling older adults. This intervention was followed by Berg's Card Sorting Test (BCST) to assess executive function domains related to task switching. The findings demonstrated that the intervention significantly increased response accuracy compared to the control group [130]. In another similar study, following the VRADA (VR Exercise App for Dementia and AD Patients) intervention, which focused on the physical and cognitive capabilities of elderly individuals with MCI, postintervention results revealed a significant enhancement in executive functions and CF compared to the control group, as measured by TMT‐B [129].

Moreover, aerobic exercise synergistically enhances the rehabilitative benefits of VR‐induced CF. Specifically, this combined protocol improves selective attention and CF indexes of the WCST and Stroop test. Also, there is a significant interaction between condition and time for WCST preservation errors and a significant time effect across all WCST and Stroop outcomes after the intervention [128]. An innovative approach, virtual reality‐cognitive rehabilitation (VR‐CR), was administered in children with autism. After teaching them how to interact with the context and reinforcing attention to salient information, there was an improvement in contextual processing of objects and the CF task, as evaluated by the Flexible Item Selection Task (FIST) [127]. Training social behaviors with a behavioral or ToM approach is a promising therapeutic approach for enhancing CF skills in autistic subjects. VR offers a useful platform that smoothly teaches these behaviors in role‐play situations by providing a safe environment for repetition of tasks, rule learning, and generalization of learned behaviors [126]. Mental simulation of socially enriched events by role‐playing situations in VR‐based environments allows enhanced insight into other minds and engages flexible behaviors in problem‐solving, both within and across contexts [240]. Through stressful contexts, rational choices and coping strategies rely efficiently on information processing dynamics [125]. To examine this interaction, a simulation of a stressful context was developed using a VR task followed by the Trier Social Stress Test (TSST). Two immersive situations were used to evaluate spatial orientation and CF. Based on the test protocol, after giving a lecture to an audience, participants select a path among various virtual labyrinths. The first test evaluated the spatial orientation and learning talent of subjects, such as how they learned multiple background perspectives and spatial cues to recognize the accurate exit sequence. The next labyrinth evaluated the CF through the WCST execution [241]. The extent to which a TSST situation is perceived as a stressful situation negatively correlates with subjects' spatial orientation and learning capacity. Additionally, if a participant experienced the TSST as an “unexpected” perception, as a measure of mild stress reaction, their CF tends to improve [242]. Immersive VR has also been administered to analyze CF, problem‐solving, and coping capacity with life problems in a patient with cognitive dysfunctions due to a thalamic stroke. Considering neuropsychological tests covering executive functions evaluation, the patient had remarkable frontal lobe dysfunction when left unsupervised, yet his performance on the traditional routine was normal. However, by providing the VR setting under the guidance of a trained expert, the immersive nature of the intervention improved self‐regulation, flexibility, and adaptability across multiple virtual cognitive challenges [243].

##### 7.2.1.4 The Potential Adverse Effects of VR

Although VR has potential therapeutic effects in multiple neurological conditions, it may lead to various adverse effects, such as nausea, dizziness, and disorientation, which are usually referred to as “cybersickness” [244]. Moreover, VR can induce eyestrain, headaches, and even physical injuries in mixed reality tasks where individuals might become distracted from their environment [245].

#### 7.2.2 Neurofeedback (NF)

##### 7.2.2.1 The Mechanism of NF Action

NF has garnered increasing attention as a noninvasive method to modulate the neural oscillations of the brain, aimed at exploring neural function or offering therapeutic results [246, 247]. This technique employs real‐time oscillatory information of brain activity to provide the participants with a mediator to self‐regulate specific brain waves [248]. Through continuous online feedback, individuals regulate their cognitive and emotional functions by a constant and dynamic linking between the current mental state and brain signals (Figure 6C) [249]. Compared to the traditional EEG NF, recent NF studies engage fMRI to analyze the real‐time hemodynamic function of the brain [246, 248]. Most investigations have shown that the fMRI‐NF‐guided approach is a promising, innovative intervention that alters the brain's regional procedures that correlate with the specific mental state [246, 250].

##### 7.2.2.2 Impact of NF on Cognitive Function

Many studies have explored the influence of NF on cognitive functions across various populations. Integration of cognitive training with NF significantly enhances working memory, episodic memory, and long‐term memory [251]. These improvements are particularly pronounced when near‐infrared spectroscopy (NIRS) is employed beside NF [252]. In healthy older adults and individuals with MCI, the NF modality that targets theta and sensorimotor (SMR) rhythm frequencies improves memory [253]. In a pilot study, home‐based NF training in MS patients enhanced cognitive performance and functional connectivity of neural networks related to cognitive processes [254]. In patients with TBI, the cognitive effects of low‐resolution tomography *Z*‐score NF (LZNF) and theta/beta NF modalities were compared with each other. The LZNFB protocol demonstrated significant enhancements in immediate and delayed recall, recognition memory, and selective attention. The theta/beta modality exhibited improvements in immediate memory and selective attention. Additionally, both interventions led to an improved QOL following the intervention [255]. The subsequent section reviews several studies that have examined the effect of NF on CF.

##### 7.2.2.3 Impact of NF on CF

**FIGURE 8:**
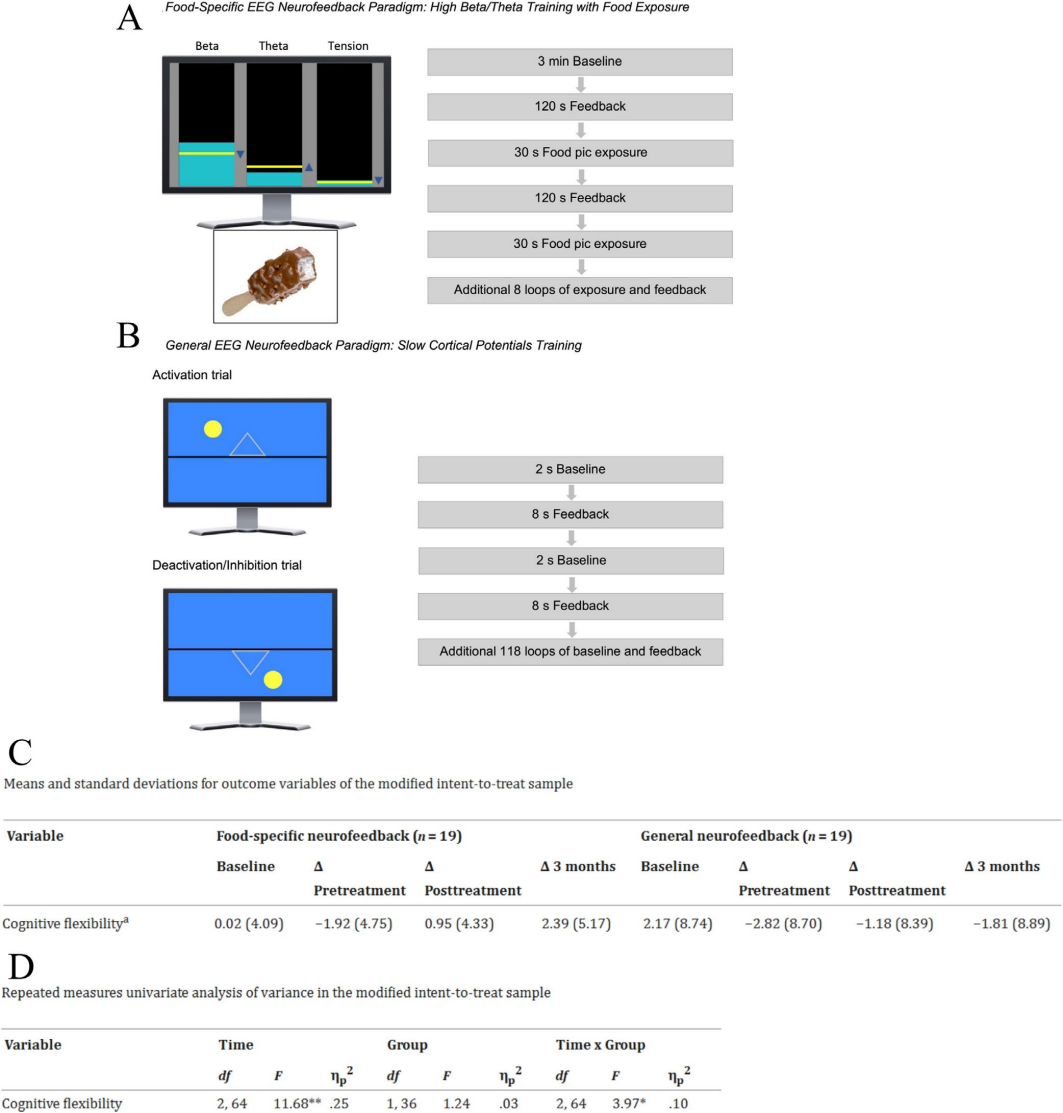
NF in patients with binge‐eating disorder. (A) Beta shows 23–28 Hz frequency band power, Theta shows 5–7 Hz frequency band power, and Tension indicates muscular contraction (60–80 Hz). Arrows represent the desired direction of training. Yellow lines indicate the threshold of the reduction/elevation reached. Turquoise bars are continuously changing and show the real‐time alterations of each frequency band's power. (B) The upward‐moving yellow ball shows the activation trial that trains cortical activation related to the electrical negativation of slow cortical potentials. The downward‐moving yellow ball shows the inhibition trial that trains cortical inhibition related to the electrical positivation of slow cortical potentials. (C, D) Neurofeedback effect on the cognitive flexibility of subjects with Binge‐eating disorder [141], Reproduced with permission.

The hemodynamic NF guided by brain imaging allows regional‐specific control and regulation of prefrontal areas, like OFC, over the activity [256], and also fNIRS could significantly inform NF training by regulating the lateral OFC activity [257]. This targeted approach enhances the accuracy and speed of switching and the reward experience associated with training [258]. In MCI patients, NF training progressively enhances beta power over training sessions, and the threshold of gaining positive feedback from the pre‐NF baseline on beta power increases significantly. Enhancement of beta wave by NF in these patients increases Central Nervous System Vital Signs (CNSVS) test scores in numerous domains, including composite memory, executive function, complex attention, reaction time, psychomotor speed, and particularly CF [136]. Following electroencephalography‐based NF on female and male adolescents with ASD for 24 sessions, CF was assessed with the set‐shifting task in the computer‐based test named Tests of Attentional Performance (TAP). After the intervention, participants had moderately better performance in emotion recognition and CF (fewer errors, faster reactions) without sex differences in reaction times or error rate [138]. In a similar study, the Flanker inhibitory control and attention test, the pattern comparison processing speed Test, the list sorting working memory test, and the DCCS test as an index of CF all showed a trend of improvement at the 2‐month follow‐up after 30 sessions of NF training over 10 weeks in children with ASD [139]. Also, a study on children with ADHD, with and without temporal lobe epilepsy, aimed to explore the effects of NF on CF and employed the TMT‐B to compare CF before and after the intervention. The results confirmed a substantial enhancement of CF in all participants, except those with mesial temporal lobe epilepsy accompanied by hippocampal atrophy [140]. In pediatric ADHD subjects with cognitive inflexibility, NF intervention trains them to alternately switch between theta up‐ and down‐regulation, which may reinforce their ability to “control their cognitive control” [247]. Abnormal EEG‐related oscillatory manifestation of the brain has been related to binge‐eating disorder (BED), and increased interest in NF as a therapeutic approach for BED [259]. A food‐based NF model decreases frontal beta power and improves theta power after regulating the cortical potentials, consequently diminishing BED‐related clinical signs, including food cravings. Besides, both general EEG NF and food‐based NF model paradigms enhance decision‐making at posttreatment duration, and CF significantly improves after food‐specific NF (Figure 8) [141]. Also, the NF has shown a promising effect in stroke rehabilitation in controlling brain activity with different protocols on cognition, particularly in the recovery of memory [260]. Repeated Theta/Beta (T/B) NF training in stroke patients with a deficiency in cognitive functions like attention and inhibition specifically improves the inhibitory control and CF score of TAP [261]. Also, Upper alpha band‐based NF training in chronic stroke rehabilitation significantly improves the memory recovery, leads to cortical “normalization” by modulating underlying pathological patterns, and particularly improves CF gain based on the flexibility subtest of the TAP [142]. In individuals with pediatric focal epilepsy after SMR NF and slow cortical potentials (SCP) NF, SMR modality reduced reaction time in the attention switching task (AST), and this reduction was positively correlated with the difference in change of theta power. Also, all groups exhibited enhanced QOL [262].

##### 7.2.2.4 The Potential Adverse Effects of NF

NF is a safe, noninvasive neuromodulatory method; however, it may cause some side effects like tiredness, headaches, mental fatigue, temporary anxiety, irritability, depression, trouble concentrating, and slower cognitive processing postintervention [263, 264]. These effects may be linked to excessive stimulation or incorrect protocols. Also, in patients with a history of epilepsy, incorrect NF settings can potentially trigger seizures [265].

## 8 Beyond the Mainstream: Other Neuromodulatory Approaches

While many studies have approved the promising effects of noninvasive neuromodulatory methods to enhance CF, there are some alternative techniques that provide therapeutic benefits on cognitive function; nevertheless, research on them remains limited. The application of these noninvasive methods may hold promise in future studies to offer suitable approaches for enhancing CF. A selection of these techniques is introduced below.

### 8.1 Transcranial Ultrasound Stimulation (TUS)

Transcranial Ultrasound Stimulation (TUS) is a noninvasive neuromodulatory treatment method that recruits focused ultrasound waves to target brain regions precisely [266]. This approach transiently enhances or suppresses regional activity, making it a promising method for addressing conditions such as depression and chronic pain, coinciding with cognitive skills improvement [266]. The findings verify TUS's effectiveness in ameliorating cognitive performance while inhibiting auditory confounds that interfere with therapeutic results [267]. For example, the closed‐loop TUS with great temporal and spatial resolution alters neural activity, resulting in long‐lasting enhancements of cognitive function and memory following its application [268]. This neuromodulatory method's potential to facilitate and inhibit neural pathways may prove its beneficial effects in future bodies of research concerning CF.

### 8.2 Transcutaneous Electrical Nerve Stimulation (TENS)

Peripheral nerve stimulation (PNS) is a method mainly used for pain relief [269]. The noninvasive form of PNS is TENS, which mildly stimulates somatosensory Aβ, Aδ, and C fibers by standard electrical current protocols through the skin [270]. Beyond pain relief, TENS can also be utilized to enhance cognitive function [271]. A study on patients with gastrointestinal tumors evaluated the results of TENS on postoperative cognitive dysfunction (POCD) symptoms. The findings verified that TENS reduced the occurrence of POCD, as indicated by higher MMSE scores of the intervention group compared to the sham group [272]. The impact of TENS on various cognitive domains, such as CF, requires further investigations that can be addressed in future studies.

### 8.3 Transcranial Magnetoacoustic Stimulation (TMAS)

Transcranial magneto‐acoustic stimulation (TMAS) is a noninvasive neuromodulation method that recruits the magnetic‐acoustic coupling to induce a low‐intensity ultrasound and electric field effect within specific brain areas [273]. In a study, the findings confirmed that TMAS enhances the cognitive function of rodents by increasing the efficiency of neural signaling in the CA1 region of the hippocampus [274]. In the rodent model of AD, after the induction of TMAS, the spatial memory of animals improved due to the decrease in hippocampal Sharp Wave Ripples (SWR) anomalies, enhancing slow gamma power during SWRs, and enhancing theta‐slow gamma phase‐amplitude coupling [275]. Considering the neuromodulatory properties of TMAS, this technique presents a promising avenue in future CF‐related studies.

### 8.4 Transcranial Alternating Current Stimulation (tACS)

Transcranial alternating current stimulation (tACS), as a noninvasive brain stimulation technique, applies a basic sinusoidal current, in contrast to tDCS, to stimulate desired and specific brain regions [276]. Prolonged application of tACS has neuroplastic effects, particularly within the theta frequency band, and may enhance cognitive function [277]. Evidence indicates that posterior theta‐tACS enhances working memory performance, whereas anterior gamma‐tACS facilitates the modulation of long‐term memory [278]. Furthermore, the cognitive outcome of tACS is closely associated with stimulation frequency. For example, offering tACS at an alpha frequency band (10 Hz), targeted at Brodmann regions F3 and P3, enhances CF in children with intellectual disabilities, evaluated by the WCST and the Clock Test [279]. Similar to tDCS, tACS may enhance CF by modifying the activity of brain areas; however, further research is required to validate this phenomenon.

### 8.5 Electroconvulsive Therapy (ECT)

Electroconvulsive Therapy (ECT) is a brain stimulation method administered under anesthesia, such that an electrical current is applied to the brain to induce a transient seizure [280]. The ECT contains an extensive therapeutic application, encompassing the management of depressive and manic episodes of unipolar and bipolar disorders, schizophrenia, PD, epilepsy, ASD, and other conditions [281]. In a clinical investigation including people with MDD, post‐ECT evaluations demonstrated substantial enhancements in the Cambridge Neuropsychological Test Automated Battery, paired association learning, and the Stockings of Cambridge. However, their pre‐ECT deficiency in intra/extradimensional set shifting remained mostly unaltered [282]. Findings indicate varying effects of ECT on cognitive performance, and the majority of investigations have focused on evaluating memory issues related to ECT [283]. Bilateral ECT may lead to enduring retrograde amnesia, and with advancing age, there may be diminished intellectual capacity and more cognitive abnormalities [284]. Beyond clinical studies, the effects of Electroconvulsive seizures (ECS) have been examined in preclinical studies. For instance, a study employed the location discrimination (LD) task to evaluate the impact of ECS on CF (reversal of cognitive strategy) and spatial pattern separation ability. ECS‐treated animals exhibited a greater number of reversals in the LD task compared to controls, regardless of the spatial separation of visual stimuli, suggesting an improved CF following ECS [285]. However, more studies are required to validate or refute the effects of ECT on CF, especially in human subjects.

## 9 Maintenance of Cognitive Gains Following Neuromodulation Approaches

Maintenance of enhancements in cognitive domains such as CF after the neuromodulatory treatment sessions requires long‐term neuroplastic synchrony and adaptations [286]. Noninvasive brain stimulation techniques like tDCS and rTMS modulate neurons' excitability and cortical networks' dynamics by specific targeting of brain regions and facilitating LTP and LTD in them [157, 287]. Similarly, NF improves the regulation of brain waves by individuals, leading to the reshaping of neural circuits over time [288]. Persistence of cognitive enhancements requires intervals between sessions, ecological validity, and cross‐context generalization to increase neural adaptations [289, 290]. Furthermore, techniques like PBM and VR have been shown to improve mitochondrial function, attentional enhancement, cognition improvement, and multimodal learning—factors known to promote synaptogenesis and structural remodeling [17, 291]. Maintenance of these treatment gains may further benefit from “booster” intervention sessions or continued engagement in cognitive tasks postintervention, aligned with the rule of “use it to keep it” [292, 293]. Generally, these neuromodulatory methods, in combination with neurobehavioral approaches, interact with intrinsic plasticity mechanisms to preserve longer‐term cognitive gains.

## 10 The Conclusion and Future Directions

**FIGURE 9:**
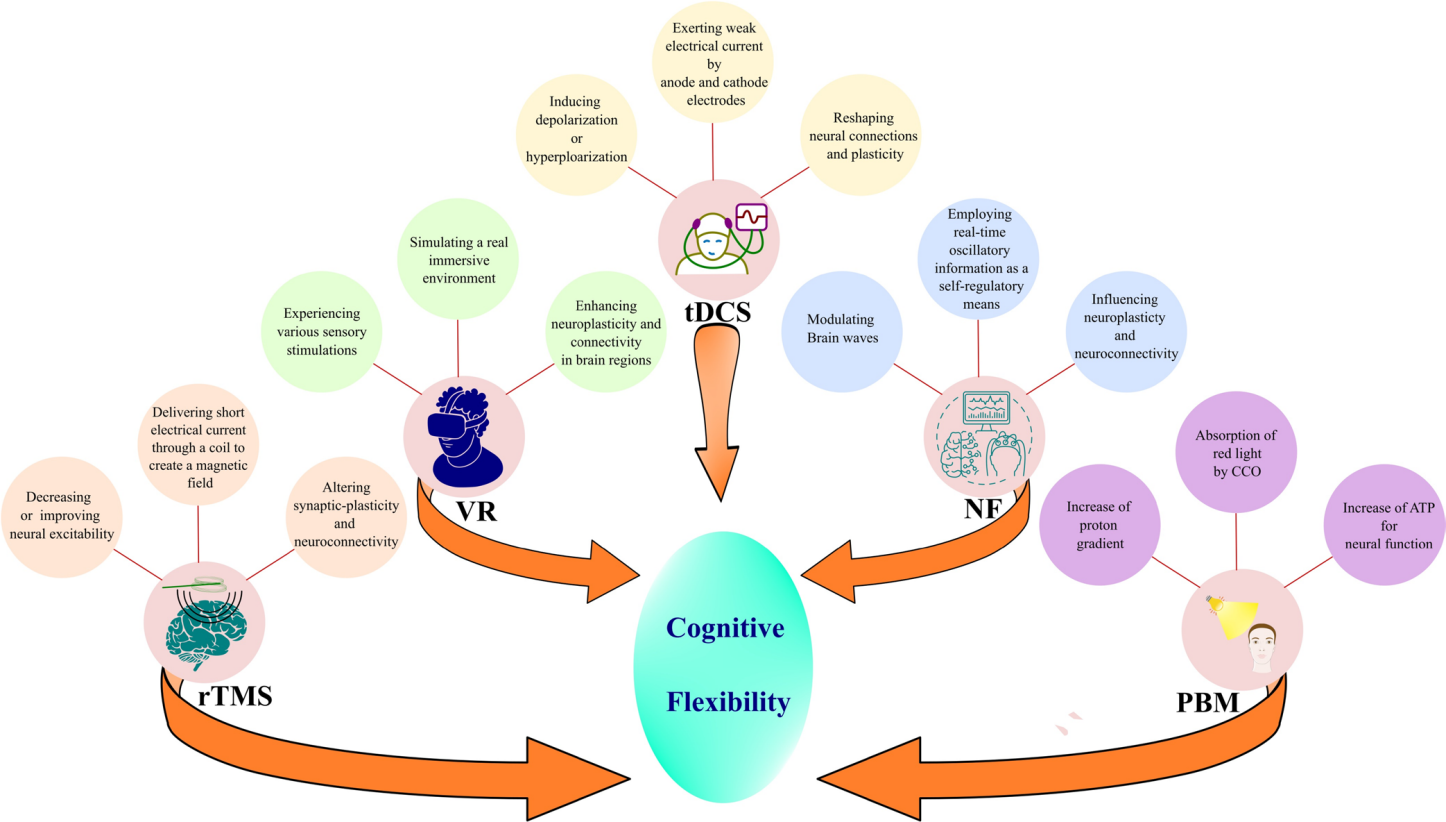
A summary of how noninvasive interventions work to improve cognitive flexibility.

CF allows individuals to modify their thoughts and behaviors in response to various environmental demands. Connections between brain areas, especially the PFC, serve as a central hub to control adaptive behaviors in the form of CF. Various neurodevelopmental and neuropsychiatric diseases induce some deficits in CF, and recent noninvasive neuromodulation methods, including VR, tDCS, rTMS, PBM, and NF, present a promising approach for improving CF (Figure 9). In the present review study, the potential of these therapeutic techniques to enhance CF and decrease the CF‐related deficits in different clinical conditions is discussed. Future studies have to emphasize developing these neuromodulation methods, comprehending the differences in response to these methods, combining various techniques, and examining the long‐lasting consequences of these interventions. Advancing these lines of inquiry holds significant promise for promoting lifelong CF and improving overall cognitive function through noninvasive neuromodulatory methods.

Although CF has been broadly studied in neuroscientific and psychological research, its practical integration into clinical neurodegenerative and neuropsychological conditions remains limited. One contributing reason to this limitation is the lack of standardized protocols that translate CF‐related scales and tasks into applicable clinical insights. Also, current diagnostic approaches for neurodegenerative conditions rarely include CF as a key criterion despite its clear relation to executive functions. Bridging this gap necessitates further complementary studies to validate CF findings across various clinical conditions and anticipate how these evaluations might complement existing diagnostic measures. Future studies in this orientation have the potential not only to enhance the clinical application of CF measures but also to support early detection and personalized interventions.

## Conflicts of Interest

The authors declare no conflicts of interest.

## Data Availability

The data that support the findings of this study are available from the corresponding author upon reasonable request.
